# Therapeutic applications of natural products in the management of venous diseases: a comprehensive review

**DOI:** 10.1007/s10787-025-01688-z

**Published:** 2025-03-12

**Authors:** Rasha E. Mostafa, Dalia E. Ali, Riham A. El-Shiekh, Ahmed N. El-Alfy, Mohamed S. Abd El Hafeez, Ahmed M. Reda, Nesrin M. Fayek

**Affiliations:** 1 Pharmacology Department, Medical Research and Clinical Studies Institute, National Research Centre, 33 El-Bohouth St., Dokki, P.O. 12622, Cairo, Egypt; 2 Pharmacognosy and Natural Products Department, Faculty of Pharmacy, Pharos University in Alexandria, Alexandria, 21648 Egypt; 3 Department of Pharmacognosy, Faculty of Pharmacy, Cairo University, Cairo, 11562 Egypt; 4 Department of Pharmacognosy, Faculty of Pharmacy, Egyptian Russian University, Cairo-Suez Road, Badr City, 11829 Egypt; 5 Department of Pharmacy, Kut University College, Al Kut, Wasit, 52001 Iraq; 6 Department of Biochemistry, Faculty of Pharmacy, Egyptian Russian University, Badr City, Cairo, Egypt

**Keywords:** Venous diseases, Treatments, Phlebotropic, Venoactive, Varicose veins, Natural products, Risk factors

## Abstract

The occurrence of venous diseases among adults is approximately 77% in females and 57% in males. These conditions are prevalent, progressive disorders that significantly affect individuals socially, physically, and psychologically, often resulting in various venous abnormalities that hinder effective blood circulation in the lower limbs. This review provides a comprehensive overview of venous diseases, focusing on their pathophysiology, symptoms, causes, risk factors, diagnosis, and complications. The symptoms associated with venous diseases are diverse and can include pain, heaviness, swelling, ulcers, and skin changes. Risk factors such as age, obesity, hormonal influences, and genetic predispositions are discussed in relation to their contribution to disease progression. The therapeutic modalities for managing venous diseases are explored, with a particular emphasis on natural products in alleviating symptoms and improving vascular health. Natural compounds, i.e., flavonoids, play a vital role in the circulatory system, supporting blood vessels and promoting healthy blood flow, in addition to their vasoprotective, antioxidant, anti-inflammatory, and anti-platelet properties. Overall, the ongoing research efforts on the efficacy of natural products will significantly enhance the management of several venous diseases in the coming years.

**Supplementary Information:**

The online version contains supplementary material available at 10.1007/s10787-025-01688-z.

## Introduction

Venous diseases are common progressive disorders which impose significant social, physical, and psychological impacts. Venous diseases are linked to significant pathological and hemodynamic changes in the lower limb veins that involve various venous anomalies significantly impairing blood return (Davies 2019). These venous anomalies result in a multiplicity of signs and symptoms which can range from mild clinical manifestations like varicose veins (VV), reticular veins, and telangiectasia to more severe ones like skin abnormalities and chronic venous leg ulcers (Costa et al. 2023).

Environmental and genetic factors inter play in the pathophysiology of venous disease leading to elevation of the ambulatory venous pressure; which significantly alters the entire structure and operation of the venous system (Youn and Lee 2019). Although any vein in the body may exhibit clinical symptoms, the lower limb venous system is frequently the most susceptible to venous diseases. The primary cause of this is the gravitational force’s greater resistance, which is noticeably greater than in other bodily parts (Alsaigh and Fukaya 2021).

Current statistics indicate that CVD affects a substantial percentage of the population, with severe forms leading to complications such as venous ulcers (Kelechi et al. 2015). Pharmacological treatments for venous diseases have evolved significantly over time, incorporating both natural and synthetic drugs, prominently featuring venoactive drugs, also known as venotonics. These medications are designed to address various pathophysiological mechanisms associated with CVD, including enhancing venous tone, reducing capillary permeability, and mitigating the release of inflammatory mediators. The efficacy of these drugs has been supported by numerous clinical trials, demonstrating their ability to improve symptoms such as pain, swelling, and heaviness in the legs (Bencsik et al. 2024). The aim of this review is to comprehensively document and analyze the various types of venous diseases, and its manifestations, including chronic venous insufficiency (CVI), varicose veins, and venous ulcers. This review will also explore the role of natural products in the management and treatment of these conditions.

### Search strategy

This review seeks to bridge gaps in current understanding and practice regarding venous diseases and their management through both conventional pharmacological means and complementary natural therapies. Scientific data on this review were gathered from various literature sources, including reputable databases such as PubMed, Google Scholar, Springer, Scopus, and Science Direct and books focused on medicinal plants. A range of keyword combinations was utilized to facilitate this research, including terms like “Venous diseases/medicinal plants,” “Phytochemicals/anti-platelets,” “Plants as venoprotective,” and “Plant secondary metabolites/vasoactive.”

### Anatomy, physiology and histology of the venous system in the lower limbs

Three major venous systems may be identified in the lower limbs: (1) superficial veins, which are mostly represented by the great and small saphenous veins, anterior accessory saphenous vein, and their branches. These veins transport blood from the skin and the subcutaneous tissues; (2) deep veins which are the key blood flow transporters; and (3) perforating veins which connect both systems (Ortega et al. 2021).

Histologically, three layers make up veins: (1) the inner layer (tunica intima) which is primarily made up of endothelial cells; (2) the middle layer (tunica media) which is made up of elastic fibers and vascular smooth muscle cells; and (3) the outer layer (tunica adventitia) which is made up of connective tissue to provide elasticity and support of the vessel (Jacobs et al. 2017). However, when venous diseases occurs, the vein’s typical structure is changed, with noticeable variations in the venous wall’s thickness and makeup (Taylor and Bordoni 2020).

Veins also contain bicuspid prolongations from the venous tissue called venous valves, which are necessary to keep blood flowing in the right direction and prevent venous reflux. In addition, to ensure unidirectional blood flow, various muscle pumps work in tandem with the venous valves. Often referred to as the “peripheral heart,” the calf muscle is particularly thought to be the most notable facilitator of venous return from the lower extremities to the heart (Tansey et al. 2019). Defects in both venous valves and calf muscle pump work lead to venous reflux and venous stasis thus causing evolution of venous diseases (Nicolaides et al. 2018).

### Classification of venous diseases

The most accurate and widely used approach for determining a precise venous disease diagnosis depend on the clinical, etiological, anatomical, and pathophysiological (CEAP) classification (Lurie et al. 2020). Table 1 summarizes the CEAP classification of venous diseases.

**Table 1:**
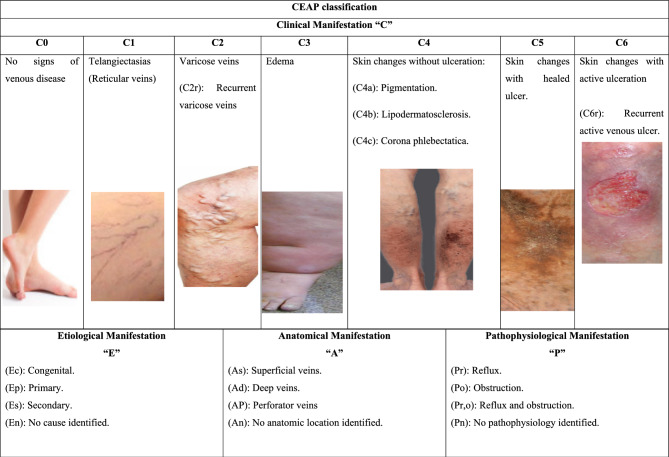
The clinical, etiological, anatomical, and pathophysiological (CEAP) classification of venous diseases

### Clinical manifestations of venous diseases

Although the precise causes of venous diseases are not fully understood, it appears that genetics and a number of proteome variations interplay in the susceptibility, onset and progression of venous diseases (Raffetto 2018).

The clinical signs and symptoms in the early stages of venous diseases are infrequent but they tend to present and worsen over time. Although VV are the most characteristic manifestation, however, reticular veins also represent a crucial clinical manifestation. A superficial injury to a big VV may cause a hemorrhage, which in certain situations may be lethal (Castro-Ferreira, et al. 2018). The most noticeable presentation of advanced venous disease is pain in the lower limbs; typically described as discomfort, pressure and heaviness that worsen at the end of the day. When venous pressure rises and lymphatic drainage is compromised, patients often exhibit malleolar edema with fovea. Other causes of the edema, such as hypoalbuminemia, hypothyroidism, or heart failure, should be ruled out by differential diagnosis (Jarošíková, et al. 2023). It is noteworthy that most patients typically notice the esthetic alteration caused by the presence of dilated superficial veins. Patients suffering venous diseases are at an increased risk of developing deep venous thrombosis (DVT), primarily in the femoropopliteal region which might cause pulmonary thromboembolism (PE). Another possible clinical indicator of venous disease is superficial venous thrombosis (SVT) which is also strongly linked to an elevated risk of developing DVT and PE (Lutsey and Zakai 2023).

Early venous disease stages usually comprise eczema and skin pigmentation, primarily caused by deposition of hemosiderin. In addition, lipodermatosclerosis, and corona phlebectatica are also linked to venous disease. Corona phlebectatica is commonly recognized as anomalous visible cutaneous blood vessels at the ankle with venous cups, telangiectasias along with capillary stasis spots (Ortega et al. 2021).

Edema and inflammation impact skin and subcutaneous tissues during advanced stages of venous diseases, ultimately resulting in the rupture of the epidermis and cutaneous ulcer formation (Nicolaides 2020).

### Etiology of venous diseases

The causes of venous diseases are multifactorial. The etiology of venous diseases is classified as per the CEAP classification into primary, secondary and congenital venous disease (Lurie et al. 2020).

#### Primary venous diseases

The primary venous disease is characterized by a progressive deterioration in the venous wall or venous valve causing improper vein dilatation and weakening; thus ultimately leading to pathological and evident venous reflux (Meissner et al. 2007a). The pathophysiology of primary venous disease includes sustained ambulatory venous hypertension that damages the vein wall and venous valves, thereby promoting the development and progression of venous disease. Primary venous disease is commonly characterized by truncal insufficiency, which occurs in the saphenous veins (Alsaigh and Fukaya 2021).

Primary venous disease is caused by a variety of genetic, environmental, occupational and life-style causes. The great majority of venous disease patients carry certain polymorphisms or genetic variations that contribute to the disease’s development. Actually, it's thought that around 17% of venous disease patients has a hereditary component (Baylis et al. 2021). The remaining percentage has been linked to a wide range of environmental elements that are typically linked to the risk factors for venous disease (Jawien 2003).

Genetic factors include familial predisposition and venous wall weakness. It is evident that there is a strong genetic component to venous diseases, particularly in conditions like VV and chronic venous insufficiency (CVI). Certain inherited disorders of the connective tissue (e.g., Ehlers-Danlos syndrome) can also predispose individuals to venous pathology. Genetic predisposition to weakened vein walls or faulty valve function increases the likelihood of venous dilation and valve incompetence (Bharath et al. 2014).

The age and gender of the patient also play important roles in the etiology and pathogenesis of venous diseases. Venous diseases are more common in older adults, as the veins lose elasticity, and the valve function deteriorates over time. Moreover, women are more likely than men to develop VV and other venous diseases, likely due to hormonal changes (e.g., pregnancy, menopause) and the higher likelihood of venous dilation in females (Robertson et al. 2008).

Environmental, occupational and life-style factors include prolonged standing or sitting as in case of occupations that involve long periods of standing or sitting which increase venous pressure, cause an inflammatory reaction in the venous wall and impair blood flow, leading to valve dysfunction and venous dilation (Elamrawy et al. 2021). Long-term standing causes a damaging and persistent venous stasis that gradually increases a person’s risk of developing venous reflux and venous insufficiency, especially when paired with other variables such aging (Bass 2007).

Obesity and increased body weight lead to higher pressure on the venous system, particularly in the lower extremities, contributing to valve failure and venous insufficiency (Davies et al. 2017). In addition, smoking is associated with a variety of venous diseases as it contributes to endothelial dysfunction and increases the risk of thrombosis (Vlajinac et al. 2012). Also, the hormonal changes during pregnancy, as well as the increased venous pressure from the growing uterus, can lead to venous dilation and VV. This is often a temporary condition but may become permanent in some cases (Ortega et al. 2022).

#### Secondary venous diseases

Secondary venous diseases are conditions that arise as a consequence of underlying or predisposing factors that alter the normal functioning of the venous system. Unlike primary venous diseases, which often occur without an identifiable cause (e.g., primary VV or CVI), secondary venous diseases result from external factors such as trauma, DVT, or external pressure on the venous system. Secondary venous disorders can cause similar symptoms to primary venous diseases, such as edema, pain, and skin changes, but their pathophysiology is driven by the underlying cause (Lurie et al. 2020). Secondary venous disease can be further classified into secondary intravenous disease, where the vein wall & valves are negatively impacted, and secondary extravenous disease, where there is no indication of vein damage but the venous hemodynamic is compromised (e.g., due to external factors like central venous hypertension, extrinsic compression from a tumor mass, previous DVT, post traumatic venous disease or in cases of limb muscle pump dysfunction) (Ortega et al. 2021).

DVT is one of the most common causes of secondary venous disease. When a blood clot forms in the deep veins, typically in the lower extremities, it causes partial or complete obstruction of venous return (Krüger-Genge et al. 2019). Over time, this can lead to post-thrombotic syndrome (PTS) or CVI, as the venous valves and vessel walls may be permanently damaged by the thrombus. If untreated, DVT can result in long-term venous stasis, valve incompetence, chronic pain, edema, skin pigmentation, and venous ulcers. The PTS is characterized by these chronic symptoms due to the damage inflicted on the veins by the clot leading to CVI with symptoms such as leg pain, swelling, heaviness, and skin changes (hyperpigmentation and lipodermatosclerosis) (Mazzolai et al. 2022).

External venous compression, either from an extrinsic mass or anatomical abnormality, can lead to secondary venous diseases. For example, tumors (such as pelvic cancers or abdominal masses), or compression due to obesity or pregnancy can obstruct venous return from the lower extremities or the pelvis (Eberhardt and Raffetto 2005).

Trauma or injury to the venous system such as those from penetrating trauma (resulting from knives ot bullets) or blunt trauma (fractures or dislocations) can lead to localized damage of the veins. This may result in venous thrombosis, venous insufficiency, or the formation of arterio-venous fistulas that alter normal venous flow. Consequently scarring or fibrosis of the veins occur resulting in CVI and long-term symptoms similar to those of primary venous diseases, such as swelling, pain, and skin changes (Stafforini and Singh 2023).

Structural abnormalities such as congenital venous malformations (CVMs) or vein walls weakened by prior surgery can result in secondary CVI. These structural defects often lead to impaired venous return and increased venous pressure, causing dilation of veins and valve incompetence (Youn and Lee 2018).

Iatrogenic causes such as surgical interventions, especially those involving the veins (e.g., vein stripping or VV surgeries), can result in secondary venous insufficiency. In some cases, iatrogenic damage to the venous system leads to scarring, stenosis, or valve dysfunction (Meissner et al. 2007b).

Obstruction of the Inferior Vena Cava can lead to secondary venous diseases, particularly in the lower extremities. Causes of Inferior Vena Cava obstruction include malignancy, thrombosis, or congenital anomalies. The result is venous congestion and impaired return of blood from the lower extremities. This condition can lead to severe edema, varicosities, and stasis dermatitis, and can result in the development of venous ulcers over time (Raju et al. 2006). It is noteworthy that secondary venous diseases generally progress faster than primary venous diseases (Labropoulos et al. 2009).

#### Congenital venous diseases

These conditions can result from abnormal development of the venous system during fetal development. The most common types of congenital venous diseases include CVMs which are clusters of abnormally formed veins that can cause a variety of symptoms depending on their size and location. These malformations result from the abnormal development of the venous system during fetal development. They are typically present at birth but may not be noticeable until later in life. Symptoms of CVMs include swelling, pain, and the formation of visible veins or a mass under the skin. Venous malformations can be located anywhere in the body but are commonly found in the legs, face, and abdomen. These malformations can grow over time, causing functional impairments or becoming prone to bleeding. In rare cases, they can cause DVT among other complications (Cooke-Barber et al. 2020).

Congenital VV, congenital CVI and congenital hemangiomas are among common congenital venous diseases. Hemangiomas are benign tumors made up of abnormal blood vessels. They are most commonly found in the skin but can also affect deeper tissues or organs. Symptoms include a rapidly growing red or purple mass under the skin, which may become painful or ulcerated. In rare cases, deep venous hemangiomas can cause more significant complications (Olsen et al. 2020).

Klippel-Trenaunay syndrome (KTS) is another rare congenital disorder caused by mutations in the PI3KCA gene. KTS is characterized by a combination of venous and capillary malformations accompanied by soft tissue or bony hypertrophy. The condition typically presents with one limb being larger than the other, with overgrowth of tissues (skin, fat, and bone), along with visible vascular malformations (Harnarayan and Harnanan 2022).

Moreover, Parkes Weber syndrome (PWS) is another congenital disease disturbing capillary, venous, arterio-venous and lymphatic systems. RASA1 gene is mutated in about 50% of PWS patients. However, about 10% of PWS patients show mutations in EPHB4 gene. It is noteworthy that PWS is commonly misdiagnosed with KTS where both cause limb overgrowth and vascular deformities (Banzic et al. 2017).

Bleb Nevus Syndrome (BRBNS) is a rare condition involving multiple venous malformations that affect the skin, gastrointestinal tract, and other internal organs. The “blue rubber blebs” are soft, blue, rubbery lumps often seen on the skin, and these venous malformations can also occur in internal organs (Agnese et al. 2010).

Other disorders associated with the development of congenital vascular diseases include cerebral autosomal-dominant arteriopathy with subcortical infarcts and lymphedema distichiasis syndrome (LDS; characterized by FOXC2 gene mutations), leukoencephalopathy (characterized by Notch3 gene mutations), Ehlers-Danlos syndrome (EDS; which is a severe congenital neutropenia characterized by changes in the G6PC3) and Chuvash Polycythemia (characterized by VHL mutations) (Fukaya et al. 2018).

### Epidemiology of venous disease

Venous diseases are common and can significantly impact quality of life. The epidemiology of venous disease varies by region, age, sex, and other demographic factors, but several general patterns and statistics provide insight into its prevalence and impact.

### Prevalence of venous disease

The prevalence of venous disease in the adult population is about 77% in women and 57% in men. The most common forms are VV & CVI, with DVT & venous ulcers being more severe manifestations (Salim, et al. 2021).

Approximately 20–25% of adults suffer from some form of VV, with a higher incidence in women. In fact, VV affect up to 40% of women and about 20–25% of men over the age of 40.

CVI affects approximately one in five adults globally. The prevalence of CVI increases with age, and it is more common in women, particularly those who have had multiple pregnancies or have a family history of venous disorders.

DVT affects 1 in 1000 individuals affected annually worldwide. However, the incidence is higher in hospitalized patients, those with cancer, and people with limited mobility or recent surgery. The overall lifetime risk of DVT is estimated at 1 in ten individuals, particularly among those over the age of 60 or those with other risk factors such as obesity, smoking, or a history of thrombosis.

It is estimated that 1–2% of people over 65 have a venous ulcer at any given time. In developed countries, venous ulcers account for about 70% of all lower extremity ulcers (Salim et al. 2021).

### Geographic and demographic variations

The prevalence of venous disease can vary across different geographic regions. Studies suggest that venous disorders may be more common in developed countries due to lifestyle factors such as sedentary behavior, higher rates of obesity, and longer life expectancy. On the other hand, venous disease may be underdiagnosed or less commonly reported in developing countries.

While venous disease is common across all racial and ethnic groups, Caucasians have the highest incidence of VV and CVI. Black and Asian populations tend to have lower rates of VV (Salim et al. 2021).

### Risk factors for venous disease

Risk factors for venous disease include age, female gender, obesity, prolonged standing or sitting, lack of physical activity, sedentary behavior, pregnancy, smoking, genetic factors and some chronic conditions like heart failure, diabetes, and inflammatory diseases (e.g., rheumatoid arthritis) (Chung and Heo 2023).

### Diagnosis of venous diseases

**Fig. 1:**
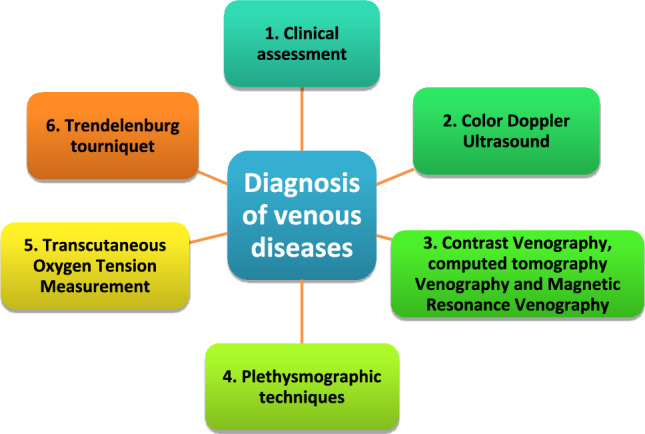
Diagnostic techniques in venous diseases

The diagnosis of venous disease typically involves a combination of clinical assessment, imaging techniques, and laboratory tests. The common techniques include:

1. Clinical assessment: A detailed patient history should be taken to identify symptoms such as pain, swelling, heaviness, fatigue, or visible varicosities. Risk factors such as prolonged standing, obesity, pregnancy, or a family history of venous disease are also considered (Barstow and Kassop 2019). Physical examination includes signs such as visible VV, leg swelling or skin changes (e.g., pigmentation, ulcers, or atrophie blanche) (Ghosh et al. 2023).
2. Color Doppler ultrasound: Duplex Ultrasound is the gold standard for diagnosing venous disease since it is non-invasive and provides real-time images of the veins. It combines traditional ultrasound imaging with Doppler flow analysis. It allows clinicians to visualize blood flow in veins, assess vein size, and detect any abnormalities such as venous reflux, venous insufficiency or thrombosis (Martinelli et al. 2021).
3. Contrast venography, computed tomography venography and Magnetic Resonance Venography are currently less commonly used due to the prevalence of Duplex Ultrasound. However, venography can be performed in some cases where a detailed view of the venous system is required where ultrasound results are inconclusive (Chen et al. 2020; Min et al. 2010).
4. Plethysmographic techniques are also non-invasive techniques which assess the severity of CVI and evaluate the function of the venous system. They identify how effectively blood is moving through the veins and whether there is venous reflux (Sannikov et al. 2020).
5. Transcutaneous Oxygen Tension Measurement is used in assessing venous ulcers and CVI via measuring the oxygen level at the skin’s surface (Leenstra et al. 2020).
6. Trendelenburg tourniquet test involves elevating the leg and then having the patient stand to observe the veins. It can help assess the function of the venous valves (Hoffmann et al. 2004). Figure 1 summarizes the diagnosis of venous diseases.

### Pathogenesis of venous diseases

The pathogenesis of venous diseases involves several interconnected processes, most notably venous hypertension, valve incompetence, and venous stasis.

Venous hypertension refers to elevated pressure in the veins, often due to a failure of the vein’s structural components or increased resistance to blood flow. This can result from incompetent venous valves; where the valves in the veins become damaged or dysfunctional and blood can flow backward (reflux) thus increasing venous pressure and promoting vein dilation Moreover, conditions like DVT can obstruct the venous flow, leading to increased pressure in the veins (Ortega et al. 2021).

Valve incompetence causes blood to flow backward, leading to blood pooling in the veins (venous reflux), which increases venous pressure and contributes to venous dilation and VV. Prolonged venous reflux leads to venous stasis, where blood pools in the lower extremities. This stasis can cause pain, swelling, and skin changes, and may eventually lead to venous ulcers. With increased pressure over time, the walls of the veins can become dilated and less elastic. This worsens the reflux and further exacerbates the venous hypertension. Over time, chronic venous hypertension can lead to structural changes in the vein walls, including thickening and fibrosis, further impeding venous return (Kumar et al. 2022).

Furthermore, CVI is often associated with a low-grade inflammatory response. The increased venous pressure can cause endothelial injury, leading to local inflammation. This may contribute to further venous wall changes, such as fibrosis and thickening. Chronic venous stasis can impair the function of the endothelial cells that line the blood vessels. This dysfunction promotes thrombosis, platelet aggregation, and the release of pro-inflammatory mediators, contributing to the worsening of venous disease (Navarrete et al. 2023).

The formation of thrombus in the deep veins can lead to venous obstruction and increased pressure in the venous system. This results in venous stasis and worsening of venous insufficiency. Over time, the clot can be resolved, but it often leaves behind damage to the vein walls and valves. PTS usually occurs as a result of long-term venous obstruction following DVT. It leads to chronic pain, swelling, skin changes, and ulcers, as the venous system is no longer capable of effectively draining blood from the lower extremities (Kumar et al. 2022).

### Management and treatment of venous diseases

#### Treatment of venous diseases aims to alleviate symptoms, prevent complications, enhance prognosis and improve quality of life

The primary strategies for the treatment and management of various venous diseases include compression treatments that focus on venous hemodynamics, medications meant to manage venous insufficiency, pharmaceutical treatments meant to target certain disease pathophysiological pathways along with interventional treatments. It is noteworthy that older patients and patients with more comorbidities respond well to the various therapies they receive, even though they need more interventions than younger patients or patients who do not have any comorbidities (Pappas, et al. 2018).

Conservative management techniques include exercise, leg elevation, weight management and compression stockings. While interventional treatments are used in patients who are not responsive to conservative treatments. Interventional treatments include sclerotherapy, laser, radiofrequency ablation (RFA) and vein stripping or ligation (Orhurhu, et al. 2021).

Compression therapy raises the interstitial pressure and consequently diminishes the diameter of the superficial and deep veins causing a reduction in the venous pressure and edema while encouraging the calf muscles to contract. Most patients encounter significant improvements with minimal discomfort while using compression stockings, which are simple to use and often the first conservative strategy to take. Thigh length stockings offer the best results in terms of venous hemodynamics and volume reduction (Dissemond et al. 2018). In addition, compression stockings promote the healing of VV and CVI-related ulcers since they reduce the likelihood of recurrent ulcerations in venous disorders (Konschake et al. 2017). Nevertheless, it must be taken into consideration that patients with peripheral artery disease need to use this specific medication with caution because it may impair lower limb blood circulation and exacerbate the underlying condition. To rule out vascular involvement, which would make the use of compression stockings inappropriate for certain individuals, the ankle-brachial index should be computed (Rabe et al. 2020).

Similarly, a variety of venoactive medications can be used to pharmacologically treat venous diseases. These substances improve the inflammatory response by reducing vascular permeability. They may also, to some degree, raise vascular tone and affect platelets aggregation (Ortega et al. 2021). Acetylsalicylic acid, pentoxifylline, flavonoids, and saponins are among the pharmacological therapies. These pharmaceutical medicines are primarily used to treat mild symptoms like pain or early stages of edema. Furthermore, there is a moderate amount of data to support their use in treating specific clinical indications of CVI, such as trophic abnormalities, cramping, restless legs, edema, and paresthesia; nevertheless, there is conflicting information about their effectiveness in treating venous ulcerations (Perrin and Ramelet 2011).

However, some medications, including sulodexide or flavonoids as diosmin and hesperidin, seem to be of promise in treating venous ulcers and CVI, especially when used in conjunction with compression treatment (Carroll et al. 2019). Under certain restrictions, acetylsalicylic acid may be used in individuals with refractory ulcers. Further research is necessary to fully understand the role of these medications (Lichota et al. 2019). Anticoagulants have been suggested for use in additional venous disease situations, including venous ulcerations, SVT, DVT and PE (Lutsey and Zakai 2023).

Several surgical techniques and technological advancements have been approved as interventional therapies for the treatment of venous diseases. These techniques are used for the efficient control of the disease, avoidance of long-term consequences and targeting optimum cosmetic outcomes (Wittens et al. 2015).

VV treatment is the most common interventional procedure for patients with venous disease. One of the most widely used methods for managing VV in the lower limbs is ultrasound-guided sclerotherapy. This technique involves using various chemical agents, such as polidocanol, sodium tetradecyl sulfate, and glycerin, to treat veins and venules. Sclerotherapy is particularly beneficial for patients with existing health conditions where more invasive treatments may not be suitable. It can also serve as an alternative to other methods for those with advanced CVI and saphenous vein dysfunction who are not candidates for surgical intervention. The effectiveness of sclerotherapy in managing venous disease symptoms is comparable to other interventions, with a low incidence of adverse effects. In addition, it has shown notable benefits for patients with superficial venous reflux, aiding in ulcer healing and preventing recurrences at rates similar to other procedures (Wong et al. 2023).

Recent advancements have introduced sclerosant foam-based and catheter-based treatments, which offer comparable results to thermal ablation techniques (de-Abreu et al. 2017).

In contrast, open surgery (such as saphenous junction ligation and stripping) has shown mixed results regarding recurrence over the mid-to-long term. Some studies suggest that surgery is more effective than conservative treatments in addressing symptoms of VV. Before endovenous thermal or chemical ablation techniques emerged, surgery was considered the standard treatment for CVI. However, due to its invasiveness and higher complication rates, including hematomas, surgical wound infections, and nerve damage, surgery is now typically reserved for patients with large, tortuous superficial vein dilations or certain vascular malformations (De Maeseneer et al. 2022).

Vein stripping or ligation is a surgical procedure used to treat VV, particularly when more conservative or less invasive treatments are not effective. The procedure involves tying off (ligation) and removing (stripping) the affected veins. The goal is to eliminate the problematic veins, which may be causing symptoms like swelling, pain, and discomfort (Gao et al. 2022).

Today, ultrasound-guided endovenous ablation has largely replaced traditional surgical approaches in the treatment of venous disease. Two primary types of endovenous thermal ablation are used: endovenous laser ablation (EVLA) and RFA. Both procedures cause controlled thermal injury to the VV, leading to clot formation and eventual fibrosis of the saphenous vein. These methods provide similar outcomes to surgery but with less recovery time and a significantly lower risk of complications. Serious complications like DVT, SVT, skin pigmentation changes and nerve damage are rare (Cai et al. 2023).

More recently, cyanoacrylate-based treatments have emerged as an alternative, although their cost-effectiveness may not be ideal in many cases. For deep vein occlusions or obstructions, particularly in acute DVT or PTS, therapeutic options like endovenous percutaneous transluminal angioplasty (PTA) with stenting are available. Stents are primarily used in patients with chronic iliac vein occlusions that have not responded to medical therapy (Ilie-Ene et al. 2023).

### Preventive measures and lifestyle changes

Prevention of venous diseases is key, especially in individuals at high risk (e.g., those with a family history, pregnant women, or those with prolonged immobility). Some preventive strategies include regular exercise, avoidance of prolonged standing or sitting, weight management and leg elevation to improve venous return (Todd 2012).

**Fig. 2:**
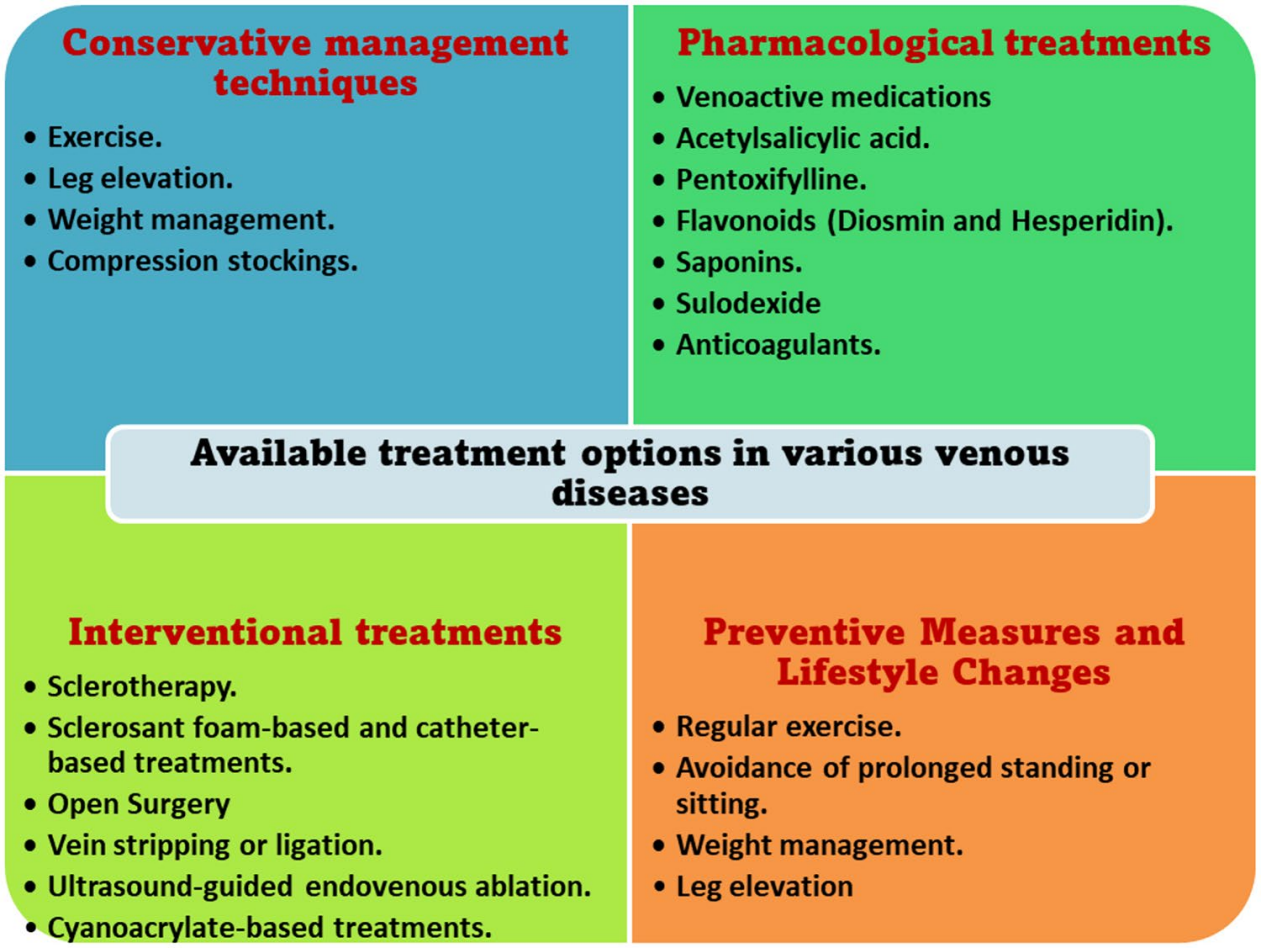
Available treatment options in various venous diseases

In summary, there is a variety of therapeutic options available for managing cardiovascular disease (CVD), each aimed at improving symptoms and alleviating the clinical impact of this chronic condition (Fig. 2).

### Future directions for the management of venous diseases

The treatment of venous disease is continually evolving, with advancements focusing on improving patient outcomes, reducing recovery times, and minimizing complications. Future directions in the treatment of venous disease are likely to revolve around several key areas including:

#### Minimally invasive and non-surgical techniques

EVLA and RFA are already standard treatments, but future developments may enhance their effectiveness and precision. Newer technologies and improvements in catheter design may make these treatments even less invasive and more comfortable for patients (Cai et al. 2023).

#### Gene and cell-based therapies

In the future, gene therapies might be used to target the underlying causes of venous disease, such as venous wall weakness or valve dysfunction. Introducing specific genes that promote the repair of damaged veins or encourage the production of proteins that support vein structure could offer novel, long-term solutions. Stem cell therapy has the potential to regenerate damaged tissues, including the endothelial lining of veins. Stem cell injections could help restore vein function in patients with CVI or PTS, leading to improved circulation and reduced symptoms (Bashor et al. 2022).

#### Personalized treatment approaches

Genomic profiling may enable clinicians to tailor venous disease treatments based on an individual’s genetic makeup. This approach could identify the most effective therapies for each patient, improving outcomes and minimizing side effects.

Artificial intelligence (AI) and imaging could be used to enhance diagnostic imaging and predict patient responses to various treatments. Real-time imaging technologies, coupled with AI algorithms, may provide more precise mapping of venous structures and better guidance for interventional procedures (Li et al. 2023).

#### Innovative biomaterials and drug delivery systems

New materials for vein stents, catheters, and other devices may enhance the treatment of venous disease by promoting better integration with the vein wall, reducing complications and prevent vein re-occlusion or reduce inflammation. Targeted drug delivery involves the targeted delivery of drugs to the affected veins, which might help in reducing the need for systemic treatments and minimize side effects. For example, drugs that promote collagen formation or improve vein elasticity could be directly applied to diseased veins (Wang et al. 2022).

#### Advanced biological and pharmacological treatments

Research into drugs that improve venous tone, reduce venous reflux, and support venous wall integrity may play a key role in the treatment of venous disease. Agents that target the underlying pathophysiology, such as endothelial dysfunction, could become part of a multimodal approach alongside procedural treatments. Regenerative Medicine use growth factors, cytokines, and other biologic agents to regenerate vein tissues and restore valve function in VV (Aleksandrowicz et al. 2021).

6. Improved long-term outcomes and recurrence prevention.

Integrated multimodal approaches such as endovenous ablation followed by sclerotherapy or the use of pharmacological agents post-procedure. This multimodal approach could help reduce recurrence rates and improve long-term outcomes. Improved patient Monitoring via wearable technology could allow for continuous monitoring of venous disease, helping to track symptoms, such as swelling or pain, in real-time. This could enable early intervention to prevent complications and reduce the risk of recurrence (Jayaraj et al. 2021).

#### Regenerative medicine

Vein regeneration focus on regenerating damaged veins or improving the function of venous valves. Tissue engineering approaches could help rebuild or replace damaged veins, providing a long-term solution for patients with severe chronic venous insufficiency (Fernández-Colino and Jockenhoevel 2021).

In conclusion, the future of venous disease treatment is poised to be shaped by technological innovations, personalized approaches, and new therapies that address both the symptoms and underlying causes of the disease. These advancements hold the potential to improve patient care, reduce the invasiveness of treatments, and ultimately provide more effective, long-lasting solutions.

### Key plants used for venous diseases

#### Blood clots, deep vein thrombosis (DVT), and superficial thrombophlebitis (SVT)

Blood clots, particularly DVT and SVT, represent significant health concerns due to their potential complications, including PE (Saha et al. 2016). DVT occurs when blood clots form in the deep veins, primarily in the legs, and is a major contributor to morbidity and mortality worldwide, with an estimated incidence of 1 in 1000 people annually (Wolberg et al. 2015). SVT involves inflammation of superficial veins, which can lead to DVT in some cases (Williams et al. 2020). Natural products have emerged as promising alternatives in the management of blood clots **(**Table 2**)**. These conditions are often linked to cardiovascular diseases, which are a leading cause of mortality globally. Natural compounds, particularly those derived from plants, exhibit antithrombotic properties that can mitigate the risks associated with traditional therapies, which often come with significant side effects. Antiplatelet, anticoagulant, and fibrinolytic activities are the main mechanisms that reflect the efficacy and potential of these natural products in addressing thrombotic disorders (Mazumder et al. 2022; Fernández-Rojas et al. 2022; Islam et al. 2016). Unlike conventional antithrombotic drugs, many natural products exhibit minimal side effects, making them safer alternatives for long-term use (Mazumder et al. 2022). So, continued exploration of natural products is essential for identifying new antithrombotic agents that can complement or replace existing therapies.

**Table 2:** Summary of the main natural used for blood clots, deep vein thrombosis (DVT), and superficial thrombophlebitis

No	Plant name	Type of extract	Part used	Family	Methods	Results	Disease	Ref
1	*Ginkgo biloba*	Ethanol	Leaves	Ginkgoaceae	Clinical trials, lab studies	Improves circulation, decreases platelet aggregation, and blood clotting	Blood clots, DVT, thrombophlebitis	(Li et al. 2019a)
2	*Aesculus hippocastanum*	Water	Seeds	Sapindaceae	Clinical trials	Reduces swelling, strengthens veins and improves venous tone in CVI	Chronic venous insufficiency	(Bencsik et al. 2024)
3	*Curcuma longa*	Ethanol	Rhizomes	Zingiberaceae	Clinical trials, animal studies	Curcumin has anti-coagulant and anti-inflammatory effects	Blood clots, DVT	(McEwen 2015)
4	*Centella Asiatica*	Water	Leaves	Apiaceae	Clinical trials, lab studies	Improves blood vessel integrity and relieves symptoms of varicose veins	Chronic venous insufficiency	(Udombhornprabha 2018)
5	*Capsicum annuum*	Ethanol	Fruit	Solanaceae	Animal studies, clinical trials	Increases blood flow, dilates vascular structures, and may even prevent coagulation	Blood circulation, varicose veins	(Rhone et al. 2018)
6	*Taraxacum officinale*	Water	Root	Asteraceae	Animal studies, lab studies	Acts as a diuretic and improves circulation, thus relieving the symptoms of CVI	Venous circulation, inflammation	(López-Pérez et al. 2022)
7	*Trifolium pratense*	Methanol	Flower heads	Fabaceae	Clinical trials, lab studies	Promotes circulation and tones up blood vessels; good for varicose veins and venous insufficiency	Blood circulation, varicose veins	(Mokhtari et al. 2020)
8	*Vaccinium myrtillus*	Ethanol	Fruit, leaves	Ericaceae	Clinical trials, lab studies	Strengthens blood vessel walls and lessens swelling thus helping varicose veins	Venous insufficiency, clot prevention	(Sparreboom et al. 2004)
9	*Pinus pinaster*	Supercritical CO2	Bark	Pinaceae	Clinical trials, animal studies	Pycnogenol increases circulation, defends veins, and minimizes swelling	Blood clots, DVT	(Gulati 2014)
10	*Mangifera indica*	Ethanol	Leaves	Anacardiaceae	Animal Studies	Blood flow maintained; prevents clotting through the strengthening of vein walls	Blood circulation, antioxidant support	(Ain 2022)
11	*Vitis vinifera*	Ethanol	Seeds	Vitaceae	Clinical trials, lab studies	Grape seed extract has antioxidants and may prevent clot formation	Blood clots, thrombophlebitis	(Bencsik et al. 2024)
12	*Ruscus aculeatus*	Methanol	Root	Ruscaceae	Clinical trials	Reduces symptoms of chronic venous insufficiency, including swelling and leg heaviness	Blood circulation, CVI	(Bihari, et al. 2022)
13	*Fagus sylvatica*	Ethanol	Leaves	Fagaceae	Clinical studies	Improves microcirculation and strengthens veins. Used in venous insufficiency symptoms	Venous insufficiency	(Sánchez-Ferrer et al. 2023)
14	*Achillea millefolium*	Ethanol	Flower, leaves	Asteraceae	Clinical trials, lab studies	Yarrow is known to enhance circulation and reduce the risk of clotting because of its anti-inflammatory effects	Blood clots, circulation	(Bijak et al. 2016)
15	*Cinnamomum verum*	Ethanol	Bark	Lauraceae	Animal studies, clinical trials	Improves circulation and blood flow and reduces blood viscosity	Venous health, blood circulation	(Raja et al. 2020)
16	*Crocus sativus*	Ethanol	Stigma	Iridaceae	Clinical trials	Saffron has anti-coagulant properties, which reduce blood clotting	DVT, blood clots	(Chen et al. 2023)
17	*Commiphora wightii*	Ethanol	Resin	Burseraceae	Clinical trials, animal studies	Known for its anti-inflammatory properties and improving blood flow	Blood circulation, varicose veins	(Rastogi et al. 2016)
18	*Withania somnifera*	Ethanol	Root	Solanaceae	Clinical studies	Enhances blood flow and exhibits slight anticoagulant properties, thereby supporting overall cardiovascular well-being	DVT, blood clots	(Basudkar et al. 2024)
19	*Eleutherococcus senticosus*	Ethanol	Root	Araliaceae	Clinical studies	Improves circulation and strengthens vein walls—helpful in cases of varicose veins and DVT	CVI, blood circulation	(Han et al. 2021)
20	*Allium sativum*	Ethanol	Bulb	Amaryllidaceae	Clinical trials, lab studies	Garlic improves circulation and has anti-clotting activity	Blood clots, varicose veins	(Alaraky 2018)
21	*Boswellia serrata*	Chloroform	Resin	Burseraceae	Clinical studies	Anti-inflammatory and blood circulation-enhancing effects	Blood clots, thrombophlebitis	(Valente et al. 2024)
22	*Crataegus oxyacantha*	Ethanol	Berries, leaves	Rosaceae	Clinical trials, lab studies	Hawthorn increases circulation and stabilizes blood vessels, thereby minimizing varicose veins and formation of clots	Blood circulation, venous health	(Nabavi et al. 2015)
23	*Cinnamomum cassia*	Ethanol	Bark	Lauraceae	Clinical studies	It improves circulation and possesses some anticoagulant properties	DVT, blood clots	(Chase et al. 2022)
24	*Piper nigrum*	Ethanol	Fruit	Piperaceae	Clinical studies	Improves circulation and may reduce the risk of clotting	Venous circulation	(Jain et al. 2023)
25	*Echinacea purpurea*	Ethanol	Root	Asteraceae	Clinical studies	Stimulates circulation and improves immune function, reduces swelling of veins	Varicose veins, circulation	(Lamponi 2021)
26	*Zingiber officinale*	Ethanol	Rhizomes	Zingiberaceae	Clinical trials, lab studies	It has good properties in improving circulation, ant-inflammatory, and blood-thinning	Blood circulation, venous health	(McEwen 2015)
27	*Salvia miltiorrhiza*	Methanol	Root	Lamiaceae	Clinical studies	Known to enhance circulatory dynamics and relieve venous edema and pain	CVI, varicose veins	(Cao et al. 2015)
28	*Mentha piperita*	Ethanol	Leaves	Lamiaceae	Clinical Studies	Peppermint can also help improve circulation and might reduce swelling and pain in the veins	Varicose Veins, Blood Circulation	(Leite et al. 2022)
29	*Alchemilla vulgaris*	Ethanol	Leaves	Rosaceae	Clinical Trials, Lab Studies	Known for its astringent and anti-inflammatory properties, it is beneficial for venous issues like thrombophlebitis	DVT, thrombophlebitis	(Radović et al. 2022)
30	*Carica papaya*	Ethanol	Leaves	Caricaceae	Clinical Studies	Improves circulation and reduces swelling associated with venous disease	Blood circulation, CVI	(Koehler, et al. 2022)
31	*Citrus sinensis*	Ethanol	Peel, fruit	Rutaceae	Clinical Trials, Animal Studies	Orange peel, which is high in flavonoids and antioxidants, enhances blood flow and decreases venous swelling	Blood circulation, varicose veins	(Leite et al. 2022)
32	*Carthamus tinctorius*	Ethanol	Flowers	Asteraceae	Clinical Studies	Reduces inflammation and supports proper blood circulation; used commonly for venous insufficiency	Venous health, circulation	(Ding et al. 2015)
33	*Hypericum perforatum*	Ethanol	Flowers	Hypericaceae	Clinical trials, animal studies	Known for its anti-inflammatory and mild anticoagulant properties, which help in blood flow and prevent clots	Venous circulation, blood clots	(Scholz et al. 2021)
34	*Silybum marianum*	Ethanol	Seed	Asteraceae	Clinical studies	Improves liver health and circulation, reducing symptoms of venous insufficiency	Venous health, circulation	(Lichota et al. 2020)
35	*Zea mays*	Ethanol	Stigma	Poaceae	Clinical trials, animal studies	Corn silk is claimed to reduce swelling and promote circulation in conditions such as DVT and varicose veins	Blood circulation, inflammation	(Raina, et al. 1997)
36	*Tilia cordata*	Ethanol	Flowers, Leaves	Malvaceae	Clinical studies	Linden has mild anticoagulant effects, therefore improves blood flow and lowers blood pressure	Venous circulation	(Risto et al. 2024)
37	*Carya ovata*	Hexane	Bark	Juglandaceae	Clinical trials, animal studies	Indicated to improve venous circulation and relieve symptoms related to venous insufficiency	Blood circulation, CVI	(Khidyrova and Shakhidoyatov 2002)
38	*Plumbago zeylanica*	Ethanol	Root, leaf	Plumbaginaceae	Animal studies	Historically used to improve blood circulation, it has mild anticoagulant properties	DVT, blood circulation	(Guguloth et al. 2023)
39	*Mentha spicata*	Ethanol	Leaves	Lamiaceae	Clinical studies	Known for its property to cause vasodilation that increases blood flow and reduces swelling in the lower limbs	Varicose veins, circulation	(Leite et al. 2022)
40	*Citrus aurantium*	Ethanol	Peel, fruit	Rutaceae	Animal studies, clinical trials	Bitter orange improves circulation and may prevent the formation of blood clots due to its flavonoid content	Blood clots, DVT	(Shahriar et al. 2018)
41	*Eucalyptus globulus*	Supercritical CO2	Leaves, oil	Myrtaceae	Animal studies	Known to improve blood flow and reduce swelling due to its anti-inflammatory properties	Blood circulation, DVT	(DiGiorgio et al. 2017)
42	*Rheum palmatum*	Ethanol	Root	Polygonaceae	Clinical studies	Reduces inflammation, improves healthy blood circulation, and may be beneficial in preventing the formation of venous thrombus	Venous health, circulation	(Yang et al. 2017)
43	*Glycyrrhiza glabra*	Ethanol	Root	Fabaceae	Clinical trials, animal studies	Licorice has an anti-inflammatory action that reduces swelling and enhances venous circulation	DVT, thrombophlebitis	(Shi et al. 2020)
44	*Cichorium intybus*	Ethanol	Root	Asteraceae	Clinical trials, animal studies	Promotes blood flow, reduces swelling, and is good for the treatment of varicose veins	Varicose veins, blood circulation	(Khosropanah et al. 2023)
45	*Cucurbita pepo*	Ethanol	Seed, fruit	Cucurbitaceae	Animal studies, clinical trials	Rich in antioxidants, pumpkin improves circulation and may help reduce venous inflammation	Blood circulation, DVT	(Harenberg 2008)
46	*Aloe vera*	Water	Leaf gel	Asphodelaceae	Clinical studies	Aloe vera is used for its anti-inflammatory properties, which reduce swelling in the legs and improve circulation	Blood circulation, varicose veins	(Udombhornprabha 2018)
47	*Rosmarinus officinalis*	Ethanol	Leaves, Oil	Lamiaceae	Clinical trials, animal studies	Rosemary “Improves blood circulation and can lower clotting,” thanks to its mild anti-coagulant properties	Blood clots, DVT	(Bencsik et al. 2024)
48	*Vitex agnus-castus*	Ethanol	Berries, Leaves	Verbenaceae	Clinical studies	Supports venous health by promoting greater blood flow and reducing inflammation in the vascular system	Venous health, circulation	(Shukhman and Bernstein 2024)
49	*Fucus vesiculosus*	Ethanol	Seaweed	Fucaceae	Clinical trials, animal studies	May help improve blood circulation and support venous health due to its iodine and antioxidant properties	Blood circulation, varicose veins	(Chandika et al. 2022)
50	*Butea monosperma*	Ethanol	Bark, leaves	Fabaceae	Animal studies, laboratory research	To increase the circulation of blood, minimize edema, and thrombi formation	Blood clots, DVT	(Jarald et al. 2009)

#### Chronic venous insufficiency (CVI)

CVI is a prevalent global pathology affecting 20–80% of the population worldwide and various factors influence it including hereditary and risk factors (i.e. obesity with BMI > 30 kg m^−2^, prolonged sitting or standing, physical inactivity, smoking, pregnancy and hormone therapy) (Krizanova et al. 2024). CVD affects all veins in the body, but lower extremity veins are most susceptible due to higher gravitational pressure compared to other regions (Kienzl et al. 2024).

Venous dysfunction, originating from venous hypertension, causes venous reflux, blood pooling, hypoxia, inflammation, and venous ulcers. CVI is also linked to severe complications like venous leg ulcers, which are more common in older patients and recur within three months (Krizanova et al. 2024).

**Fig. 3:**
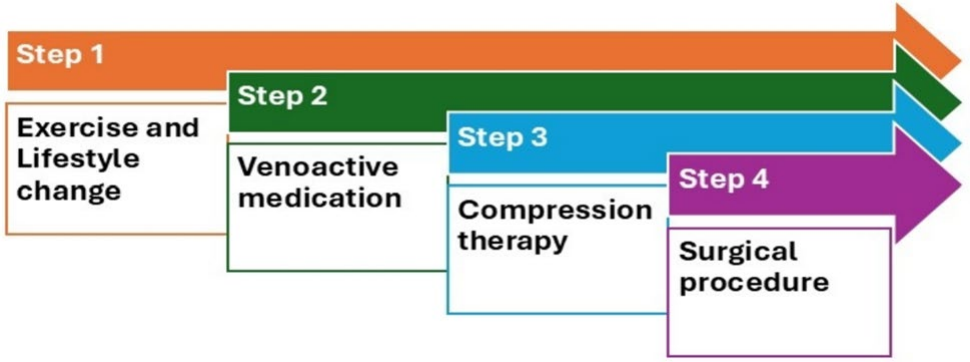
Steps for CVD therapy as based on the individual patient’s symptoms and severity

The three fundamental modalities of conservative management for CVD are exercise and lifestyle (focusing on enhancing lower limb muscle strength, ankle flexibility, and physiotherapy, quitting smoking and reducing excessive alcohol consumption), compression therapy (utilizing elastic stockings or wraps on the legs, ankles, and feet to avert blood pooling and fluid accumulation), and pharmacological intervention (administering venoactive agents, including both natural and synthetic compounds such as butcher’s broom (*Ruscus* spp.) extract, micronized purified flavonoid fraction (MPFF), calcium dobesilate, horse chestnut (*Aesculus hippocastanum* L.) extract, hydroxyethyl rutosides (troxerutin, troxerutin), common grape (*Vitis vinifera* L.) extract, and sulodexide) (Bencsik et al. 2024). The pharmacist recommends conservative CVD therapy based on the individual patient’s symptoms and severity, following steps 1–4 **(**Fig. 3**)**. In severe cases, Step 4; surgical invention i.e., removal of veins or vein sections that have lost their function, is the responsibility of a physician.

Certain medicinal plants have been found to alleviate symptoms of CVD and delay the progression of vessel wall impairment or destruction **(**Table 3**)** (Bencsik et al. 2024). Flavonoids, classified as medical food, are found to be the most effective treatment methods for CVI alongside compression treatment and exercise, despite various research methods being explored (Krizanova et al. 2024). Phlebotonics, a group of plant flavonoids, are considered venoactive and vasoprotective agents. They have varied opinions, with some studies showing benefits like diosmin (MPFF) for reducing chronic venous disease symptoms and slightly reducing edema and ankle circumference (Krizanova et al. 2024).

**Table 3:** Summary of the main natural venoactive drugs for chronic venous disease; constituents, treatment duration, effects on symptoms, mechanism of action, possible side effects (Bencsik et al. 2024)

Plant name	Common name	Family	Geographical origin	Part used	Active constituents	Duration for treatment	Relieve symptoms of	Mechanism of action	Possible side effects	Reference
*Aesculus hippocastanum* L	Horse chestnut	Sapindaceae	Asia	Seeds	Acylated triterpene saponins: aescin 3–10% (= protoaescigenin + barringtogenol C)	At least 4 weeks of treatment	Discomfort and heaviness of legs related to minor venous circulatory disturbances and signs of bruises, such as local edema and haematoma	Decrease the release of inflammatory mediators (e.g. cytokines, arachidonic acid metabolites such as prostaglandins and leukotrienes) and block elastase and hyaluronidase activities in the process of degradation of connective tissue	Skin inflammation, thrombophlebitis, subcutaneous induration, severe pain, ulcers, sudden swelling of one or both legs, and cardiac or renal insufficiency	(Bencsik et al. 2024)
*Vitis vinifera* L	Red grape vine	Vitaceae		Leaves and seeds	polyphenols (different flavonoids such as flavonol-glycosides and glucuronides, catechins, epicatechins (both monomers and dimers), and anthocyanidins)	12 weeks	Relieve symptoms of discomfort and heaviness of legs related to minor venous circulatory disturbances, (ii) symptomatic relief of itching and burning associated with hemorrhoids, and (iii) symptomatic treatment of cutaneous capillary fragility	Improve microcirculation, and oxygen supply, and significantly reduce edema, leg pain, heaviness, and tension	Hypersensitivity reactions of the skin (itching, erythema, urticaria), nausea, gastrointestinal complaints, and headache	
*Ginkgo biloba* L	Maidenhair tree	Ginkgoaceae	China	leaves	Flavonols (quercetin, kaempferol, isorhamnetin, and their mono-, di-, and triglycosides), Biflavonoids, (amentoflavone, ginkgetin, bilobetin, sciadopitysin) and Terpene lactones (diterpenes, e.g. ginkgolides A, B, C, D, K, L, M, N, P, Q and bilobalide)		Used in combination with troxerutine and heptaminol, to protect against endothelial injury occurring during ischemic or hypoxic periods of chronic venous insufficiency		Gastrointestinal disorders (diarrhea, abdominal pain, nausea, vomiting), headaches, dizziness, and allergic reactions	
*Melilotus officinalis* (L.)	Melilot or yellow sweet clover	Fabaceae	USA and Canada	Aerial parts	Coumarins (scopoletin, umbelliferone, melilotin). *o*-hydroxycinnamic acid derivative (melilotoside), Dicoumarols, Flavonoids (flavonols, e.g. quercetin, kaempferol), and triterpene saponins (melilotoside A, B, and C)	1 or 2 weeks	Relieve symptoms of discomfort and heaviness of legs related to minor venous circulatory disturbances”, and “for the treatment of minor skin inflammations. Diminution of the sovrafascial edema	Dicoumarol inhibits the reduction of vitamin K and thus prevents the gamma-carboxylation of the glutamate residues in clotting factors II, VII, IX, and X Coumarins stimulate proteolysis in the tissues affected by chronic lymphedema	Allergic skin reactions	
*Rosmarinus officinalis* L		Lamiaceae		Leaves	Monoterpenes such as 1,8-cineol, α-pinene, camphor, bornyl acetate, borneol, camphene, α-terpineol and ( +)-verbenone. Also, abietane diterpenes, triterpenes, flavonoids and rosmarinic acid		Relief of dyspepsia and mild spasmodic disorders of the gastrointestinal tract. Relief of minor muscular and articular pain and in minor peripheral circulatory disorders	Treat phlebitis related to intravenous therapy and increase local circulation and cause heat and relief of pain and inflammation	Lack of adequate data	
*Ruscus aculeatus* L	Butcher’s broom	Asparagaceae	Europe	Leaves	Steroidal saponins such as ruscoside and ruscin	2 weeks	Relieve the symptoms of discomfort and heaviness of legs related to minor venous circulatory disturbances. Also, relief of itching and burning associated with hemorrhoids	Decreases capillary filtration rate in healthy volunteers and patients with CVD. Also, it reduces vascular permeability Ruscogenins has antielastase activity. Ruscus extract could significantly decrease the venous diameter in deep veins and at the same time led to an increase in flow parameters	Inflammation of the skin or subcutaneous induration, ulcers, sudden swelling of one or both legs, or cardiac or renal insufficiencies In case of hemorrhoids, rectal bleeding occurs	
Vaccinium species	Bilberry Cranberry Blueberry	Ericaceae	Eurasia	Fruit	Anthocyanins, expressed as cyanidin 3-O glucoside Proanthocyanidins, apigenin, luteolin, chrysoeryiol, kaempferol, quercetin, and their glycosides, triterpenic and phenolic acids, and vitamins B 1, B 3, B 5, and C	8 weeks	Relieve symptoms of discomfort and heaviness of legs, symptoms of cutaneous capillary fragility related to minor venous circulatory disturbances		nausea, vomiting, diarrhea, constipation, and dyspepsia	
*Centella asiatica* (L.)	(gotu kola	Umbelli ferae (Apiaceae)	India	Aerial parts	Triterpene saponins, asiaticosides, madecasos side (brahminoside), asiatic acid, and madecassic acid (brahmic acid)	Three months	Prevention and treatment of CVD, including post-thrombotic syndrome aid in the healing of minor wounds, but it is exclusively based upon long-standing use	Strengthening of the weakened veins. Moreover, it can stimulate the formation of hyaluronidase and chondroitin sulfate and exert a balancing effect on the connective tissue. Its final beneficial effect in CVD seems to be mostly the improvement in microcirculation [122]. Besides that, it can decrease the capillary filtration rate by improving microcirculatory parameters	Hypersensitivity may occur to the active substance, or excessive oral intake of C. asiatica can cause headache and transient unconsciousness	

In addition, some secondary metabolites, such as triterpene saponins, exhibit beneficial effects on vascular walls, but their mechanism differs, involving inhibition of enzymes responsible for vein wall structure breakdown (Bencsik et al. 2024).

Other medicinal plants (*Ruscus aculeatus* L., *Aesculus hippocastanum* L., *Centella asiatica* (L.) Urb.) and their active compounds (ruscoside, aescin, asiaticoside) besides *Ginkgonis folium*, *Meliloti herba*, *Rosmarini aetheroleum*, *Rusci rhizoma*, and *Myrtilli fructus recens* also have important places (Bencsik et al. 2024).

#### Varicose and spider veins

VV, also known as varicosities, are enlarged, and twisted, causing backward flow and turbulence in blood circulation, damaging legs (Khan et al. 2024). VV can be classified as spider veins (angioectasis; telangiectases), thread veins, or matted veins (Gawande et al. 2024). They can cause blood reflux, venous hypertension, and swelling. Risk factors include gender (more occurrence in women), family history, prolonged standing, older age, hormonal Changes (i.e. puberty, pregnancy, multiparous and menopause, post-menopausal, hormone replacement, and other medicines containing estrogen and progesterone), lack of physical activity, obesity, alcohol, and smoking (Khan et al. 2024).

Some of the considerations that may guide the choice of treatment include 1) physical therapy: exercise, yoga and massage, 2) compression therapy: using the special type of compression stockings, 3) non-surgical treatment: including sclerotherapy (via injecting sclerosing agents such as sodium salicylate, polidacanol, chroamted glycine using small needles, and this treatment is accompanied by compression stockings to be worn after the sclerotherapy, laser treatment (sending strong bursts of light onto the vein), ultrasound guided foam sclerotherapy (damaging of the endothelial layer of the vein to create a blockage and scar formation in the dilated veins), and endothermal ablation (using energy from radiofrequency and lasers to fasten the affected veins), 4) surgical treatment: including vein stripping (inserting Of special wires onto the affected vein) and ambulatory phlebectomy (superficial veins are removed by performing incisions in the skin), 5) natural treatment via several plants (Gawande et al. 2024).

Herbal medicines (Table 4) are commonly used for VV treatment, with formulations varying based on an individual’s constitution and symptoms.

**Table 4:** Herbal medicines as used for treating varicose veins

Herb	Description	Role	Reference
Habb-e-Asgand	Referred to Ashwagandha or *Withania somnifera*	Possess strengthening qualities that could promote circulation and vascular health	(Khan et al. 2024)
Qurs-e-Zeequn Nisa	Unani preparation with various botanical constituents, including *Zingiber officinale* (ginger) and *Cyperus scariosus* (nagarmotha)	Have anti-inflammatory and circulatory properties	
Majoon Ushba	Unani herbal paste	Increase blood flow and lessen swelling. It frequently contains components like *Terminalia chebula* (Halela Siyah) and *Colchicum luteum* (Ushba)	
Horse chestnut seed extract	(*Asculus hippocastanum*; Family hippocastanaceae) contain aescin, tannins, flavonoids, quinines, sterols and some fatty acids, coumarins and scopolin Aescin is the most active constituent of the horse chestnut seeds and comprises about 16–20%	Help in toning the veins, reduce the vascular permeabilityand enhance the venous return	(Gawande et al. 2024)
Gotu kola	*Centella asiatica*; Family umbelliferae contains a chemical called triterpenic fraction of *Centella asiatica*	Reducing swelling and improving blood flow	
Apple cider vinegar	Raw grated apple extracted from fruits of *Malus pumila,* Family Rosaceae	Helped in providing relief from the pain irritation, ulceration, pigmentation, edema, cramps and itching	
Butcher broom	Root of *Ruscus aculeatu*s; Family Liliaceae contain active constituent steroidal saponins, neoruscogenin and ruscogenin	Anti-inflammatory, vasoconstriction, antihemorrhagic	
Garlic	Ripe bulbs of *Allium Sativum*; Family Liliaceae Contains allicin (volatile oil), carbohydrates, proteins, fats and mucilage	Reducing inflammation and the symptoms of varicose veins	
Amla	Fresh fruit pericarp of *Emblica officianalis* Gareth or *Phyllanthus emblica* linn. Family Euphobiaceae contains emblicanin A and B, punigluconin and pedunculagin vitamin, iron and calcium	Help improve blood circulation and reduce inflammation	
Tomato	Fruit of *Solanum lycopersicum* derived from two wild ancestor species, *Solanum pimpinellifolium* and *Solanum cerasiforme*	Cure varicose veins	
Grapes seeds extract	Seeds of *Vitis vinifera;* family Vitaceae oligomeric proanthocyanidins (OPCs)	OPCs make blood vessels more elastic and also less likely to leak fluids that cause the leg swelling often associated with varicose veins Reducing the swelling itching and pains caused due to varicose veins	
Ginger	(Rhizome (underground stem) of *Zingiber officinale;* Zingiberaceae)	Has the super ability to dissolve fibrin and reinstate blood circulation in vessels. Remember it is not an easy task to break down fibrins and it requires powerful food such as ginger to accomplish	
*Terminalia arjuna*	Bark extract of *Terminalia arjuna*	Reducing the symptoms like pain, edema, inflammation, pigmentation, induration and also expediting ulcer healing	(Gawande et al. 2024)

Leech therapy is an effective method of bloodletting for treating VV. The leech discharges its saliva, which contains bioactive chemicals, which can extract stagnant or clotted blood from the veins. This therapy helps cleanse the blood, reduce vein pressure, and congestion. The Unani system of medicine uses Irsal-e Alaq (leech therapy) and Tanqiya-e Sauda (evacuation of black bile from the body) for managing varicose veins. The treatment regimen includes Itrifal Sagheer with Zanjabeel and Joshanda Aftimoon, which effectively reduces symptoms and signs of varicose veins (Khan et al. 2024).

#### Venous ulcers

Venous ulcers, specifically venous leg ulcers, represent a significant clinical challenge as they are the most prevalent type of chronic wound, accounting for 60–80% of all leg ulcers (Franks et al. 2016). These ulcers typically arise from CVI and venous hypertension, conditions characterized by impaired venous return and elevated venous pressure (Kumar et al. 2022). Chronic inflammation plays a pivotal role in the ulcer formation process; inflammatory cells become trapped in the fibrin cuff surrounding the ulcer, leading to further tissue damage and impaired healing (Yang et al. 2024). Traditional treatment modalities include compression therapy, wound care, and surgical interventions; however, these approaches may not always yield satisfactory results. Consequently, there is growing interest in the role of natural products as adjunctive therapies in the management of venous ulcers (Table 5). Natural products have been recognized for their potential therapeutic benefits in enhancing wound-healing processes. Various phytotherapeutic agents possess anti-inflammatory, antioxidant, and antimicrobial properties that can promote tissue regeneration and improve vascular health. By integrating insights from current literature on both conventional treatments and emerging natural therapies, this document seeks to provide a comprehensive overview that may inform clinical practice and guide future research efforts in optimizing care for patients suffering from venous ulcers.

**Table 5:** Phytochemicals concerning the treatment of venous ulcer diseases

Plant	Part used	Active constituents	Method	Results	References
*Acacia arabica* (Lam.)	Gum	Arabinogalactan-proteins	*In-vitro* antimicrobial assay for the microbes that are associated with ulcer; *Bacillus licheniformis* (ATCC 14580), *Escherichia coli* (ATCC 25922), *Pseudomonas aeruginosa* (ATCC 27853) and *Staphylococcus aureus* (ATCC 25923) using hydrogel wound dressing	Gum acacia showed MIC 500 to 600 µg mL^−1^ against species known to be implicated in wound infections	(Bhatnagar et al. 2013)
*Acalypha indica*	Aerial parts	Alkaloid; Acalyphine, flavonoids; luteolin, quercetrin and kaempferol, saponins and tannins	To estimate several biochemical and biophysical studies and to examine histological modifications both with and without extract therapy, the wound tissue was excised using *in-vivo* model	Treatment reduced lipid peroxidation and oxidative stress while simultaneously raising ascorbic acid levels. In addition, it enhanced collagen synthesis	(Ganeshkumar et al. 2012)
*Achillea biebersteinii Afan*	Root	1,8-cineole, α-terpinene, camphor, borneol, piperitone, and β-Eudesmol	Different solvent extracts of studied plant evaluated against excision and incision wound in rat and mice	All extracts showed remarkable healing activity against the two types of wounds	(Akkol et al. 2011)
*Achyranthes aspera* L	Leaf	Alkaloids; Achyranthine, saponin, flavonoids; quercetin, tannins and sterols	Simple ointment from plants applied with different concentrations (2.5%, 5% and 10% (*w/w*)) to wounded albino rats and compared with reference drug 1% silver sulphadiazine	Results showed elevation of fibrocyte count, notable level of neovascularization, and epithelization especially in plant ointment with concentration of 5%&10%	(Fikru et al. 2012)
*Actinidia deliciosa* (Kiwi)	Peel	Vitamins (C and E), potassium, fiber and folic acid. Flavonoids; rutin, epicatechin and quercetin glycosides phenolic acids; ferulic acid and caffeic acid. Acids that are organic either citric, malic, or quinic	*In-vivo* study was designed to assess the effect of kiwi peel gel and its nanoformulated gel	Reduction in wound area reached 69.12% on day 12	(Bassam et al. 2024)
*Ageratina pichinchensis*	Aerial part	Bioflavonoids; Taxifolin, oligomeric procyanidins, epicatechin and catechin Phenolic acids; ferulic and caffeic acids	Thirty-four patients were divided into two groups and received *A. pichinchensis* extract and 7% propylene glycol alginate, respectively	Within 10 months of treatment, patients who received the extract showed 100% healing while the control group showed healing by 81.8%	(Romero-Cerecero et al. 2012)
*Allii bulbus, Hypericum perforatum and Calendula officinalis*	Bulb, aerial part and oil extract, respectively	Organosulfur compounds; allicin, diallyl disulfide, trisulfide and S-allyl cysteine (*A. bulbus*) Naphthodianthrones; hypericin and pseudohypericin Phloroglucinols; hyperforin Flavonoids; Quercetin, kaempferol, and rutin (*H. perforatum*) *α*-cadinol, *γ*-cadinene (*C.officinalis*)	Twenty five patients treated with herbadermal® (*A. bulbus, H. perforatum and C. officinalis*) for 7 weeks	After the treatment, the percentage of treatment was 99.1%	(Kundaković et al. 2012)
*Aloe barbadensis* (Aloe vera)	Leaf, gel	Anthraquinones; Aloin and Emodin, vitamins; B12, A, E and C, salicylic acid, amino acids and minerals	The study designed to compare the antibacterial activity of *A. vera* leaf extracts and gel with common antibiotics (methicillin, erythromycin, bacitracin, novobiocin and vancomycin) against Gram-positive (*S. aureus, Staphylococcus* epidermidis, and Streptococcus pyogenes) and Gram-negative (*P. aeruginosa*) bacteria isolated from human skin wounds, burns, and acne	Vancomycin, the most effective antibiotic, had 80.5% and 72.2% efficacy against gram positive and Gram-negative isolates, respectively, whereas *A. vera* exhibited 100% activity against gram negative bacteria and 75.3% against all tested Gram-positive isolates. In addition, *A. vera* gel shown antibacterial efficacy with MIC < 400 μg/mL against multidrug-resistant *P. aeruginosa* that isolated from burn wound infection patients	(Bashir et al. 2011)
	Gel		Clinical study, 50 patients with partial and superficial wounds divided into 2 groups (group 1 treated with Aloe vera gel and the other treated with 1% silversulphadiazine cream)	Significant pain relief and increase of epithelialization in case of aloe vera treated group	(Shahzad and Ahmed 2013)
*Camellia sinensis*	Leaf	Epigallocatechin-3-gallate	*In- vitro* study based on using chitosan green tea polyphenols complex (CGP)	(CGP) enhance granulation and epithelialization. These outcomes could be attributed to its antioxidant qualities and transglutaminase (TGM) expression activation	(Qin et al. 2013)
*Centella asiatica*	Herb	Triterpenoid saponin; Asiaticoside	*In-vivo* model on burn wound area of mice	Treatment enhance angiogenesis during skin wound healing due to the stimulation of VEGF synthesis brought on by the co-induction of MCP-1 expression in keratinocytes and IL-1β expression in macrophages by asiaticoside and MCP-1	(Kimura et al. 2008)
*Chamomilla recutita Matricaria* *chamomilla*	Flower	Isolated flavonoid; apigenin	*In-vitro and in-vivo* model used to assess the effect of apigenin on collagen and skin thickness	Apigenin activates type-I and type-III collagen synthesis by promotion of smad2/3 signaling pathway and this was approved by different techniques as PCR and western blot	(Zhang et al. 2015)
*Curcuma longa*	Rhizome	Curcumin	*In-vivo* model on wound -excised rat	Curcumin enhance re-epithelialization, collagen synthesis and decrease ROS	(Dai et al. 2009)
*Ficus racemosa*	Root	Flavonoids, alkaloids and tannin	Incision and excision models using rats were used to assess the effect of plant extract	In incision model, aqueous plant extract showed greater breaking strength, the percentage of wound contraction, and the duration of epithelialization than excision model	(Murti and Kumar 2012)
*Echinacea pallida* Nutt Or *E. purpurea*	Root	Echinacoside and echinacin	*In-vivo* study was designed to assess the effect of a gel (1% ethylcellulose) with 100 mg of *E. pallida* dried extract that applied only once, on abraded skin of rats and then the wounded area was covered with a patch. Otherwise, vehicles are used only with control group	Rats given after 48 and after 48 and 72 h, rat treated by *E. pallida* did not exhibit any symptoms of inflammation, and it was approved histologically	(Speroni et al. 2002)
Garcinia mangostana	Fruit	Xanthone, mangostins, garcinone E, tannins and flavonoid	Nanoformulation of fruit extract	This formulation showed significant acceleration of healing compared with control with anti-inflammatory and antibacterial effect	(Charernsriwilaiwat et al. 2013)
*Ginkgo biloba*	Leaf	Flavonoids; isorhamnetin, Quercetin, and kaempferol, Ginkgolides and organic acids	Excision and dead space. Wound healing effect was studied on rats using dose 50 mg/kg	Ginkgo biloba has a significant pro-healing effect, which may be due to its influence on the collagenation stage of wound healing	(Bairy and Rao 2001)
*Ganoderma praelongum* *Glycyrrhiza glabra*	Fruit Root	Terpenoids, polysaccharides, sterols and proteins Glycyrrhizin	The effects of gel formulations containing 0.3% G. praelongum, 2.5% G. glabra, and a combination of the two extracts on the stages of wound infection, wound contraction, and epithelization were evaluated on wounded mice	Combination gel had promising effect against methicillin resistant microbes (*in-vitro*). Moreover, different formulations showed considerable contraction and epithelization (*in-vivo*)	(Ameri et al. 2013)
*Hevea brasiliensis*	Rubber tree	Latex that consists of 35% cis-1,4-polyisoprene, 5% non-isoprene molecules, and 60% water	Fourteen patients were chosen to receive *Hevea brasiliensis* biomembrane treatment, and seven received Fibrase® as control drug. Over the duration of 120 days, the wound-healing index was used to track the patients’ clinical and photographic progress	After the treatment duration 42% of ulcer healing was observed	(Frade et al. 2012)
*Hibiscus rosa*	Flower	Anthocyanin, flavonoids, phenolics and polysaccharides	(5 and 10% *w/w*) plant extract were used to treat different types of wounds in rats (incision, dead-space and excision)	The results showed decreasing in the epithelization period and a rise in the dry and wet granuloma weights, tensile strength, and wound closure rate	(Bhaskar and Nithya 2012)
*Hypercium perforatum*	Oil of aerial part	Hypericin Hyperphorin	*In-vivo* model and X-ray was used to evaluate healing	Oil plant emulsion showed significant wound healing compared with mupirocin ointment that used as standard dry by percentage 97% and 68%	(Nayak et al. 2017)
*Kigelia pinnata*	Bark	Napthoquinone; Lapachol, Phenolic compounds, alkaloids, coumarins and iridoids	Rat with incision, dead-space and excision treated with oral aqueous extract (250 mg/kg and 500 mg/kg)	The bark extract had the ability to enhance hydroxyproline levels, improved granuloma breaking strength, and skin tensile strength	(Sharma et al. 2010)
*Lavandula* *angustifolia*	Flower	Essential oil; linalool and linalyl acetate	Prepared alginate nanofibers were evaluated against microorganisms and inflammatory markers that are associated with wound	Pro-inflammatory cytokines are significantly reduced when alginate-based nanofibers are applied to fibroblasts or animals. The suppression of the production of cytokines was also noticed	(Hajiali et al. 2016)
*Lawsonia inermis* (henna)	Leaf	Quinones (arbutin, lawsone and anthraquinone), flavonoids (kaempferol, apigenin, luteolin, quercetin, and catechin), terpenoids and xanthones	*In-vivo* study that designed using Wound dressings was made from oxidized starch nanofibers and henna-gelatin and assessed using immunohistochemistry	Reduction of inflammation and speed up the healing of wounds	(Hadisi et al. 2018)
*Lygodium flexuosum*	Leaf	Tannins, alkaloids, glycosides and flavonoids	Excision wound model using albino rats was used to assess different concentrations of plant extract (4 &5%) with different doses	The extract ointment had the ability to reduce the time needed for epithelization and raised the percentage of wound contraction	(Chandra et al. 2015)
*Mimosa tenuiflora* (Willd.)	Bark	Polyphenols, triterpenoidal and steroidal saponins and low quantity of alkaloids	Forty patients having a clinical diagnosis of VLU were part of the trial; twenty were randomly allocated to the experimental group that treated with extract hydrogel for 12 weeks and twenty to the control group. The study population was 61 years old on average	After the treatment, a significant decrease in ulceration areas was observed; by the eighth week, the ulcer-size reduction had reached 93% as the mean value for the group. Finally, all patients were cured at the end of the trial	(Rivera-Arce et al. 2007)
			Nineteen women and 13 men suffered from VLU were subjected to treatment with extract hydrogel	After the treatment with hydrogel for 8 weeks the healing percentage was 22%	(Lammoglia‐Ordiales et al. 2012)
*Moringa oleifera*	Seed	α-tocopherol, β-Sitosterol, flavonoids mainly (Apigenin and Naringenin), phenolic acids such as (gallic, ferrulic caffeic and vanillic) and proteins	Hydrogel wound dressing is used in an *in-vitro* antimicrobial assay for the bacteria that cause ulcers, including Bacillus licheniformis (ATCC 14580), Escherichia coli (ATCC 25922), Pseudomonas aeruginosa (ATCC 27853), and Staphylococcus aureus (ATCC 25923)	*M. oleifera* demonstrated MICs of 300–700 µg mL^−1^ against species that are known to cause wound infections	(Bhatnagar et al. 2013)
*Nigella sativa*	Seed oil	Nimbidin and thymoquinone	Wound healing was assessed using carbopol emulgel prepared from studied seed oil	Unsaturated fatty acids stimulate production of vascular endothelial growth factors that have evidenced a role in wound healing	(Thomas et al. 2024)
*Ocimum basilicum (Basil)*	Seed oil	Mucilage mainly Glucomannan and xylan	Dressing of seed mucilage with ZnO nanoparticles that crosslinked with borax was examined against bacteria associated with wound	Borax crosslinking enhanced the antibacterial dressings made from Basil Seed Mucilage (BSM) with ZnO-NP against *S. aureus and E. coli*	(Tantiwatcharothai and Prachayawarakorn 2020)
*Olea europaea*	Leaf	Secoiridoid oleuropein	Hexane and aqueous extract of the plant leaf was compared with reference ointment Madecassol® to heal linear incision and circular excision using *in-vivo* model	In comparison to the other groups, the animals treated with the aqueous extract showed an impressive rise in wound tensile strength (34.8%) on incision models and greater contraction (87.1%) on excision	(Koca et al. 2011)
*Oreochromis niloticus L*	Skin collagen	Gel from collagen extract	Cutaneous wound-healing process in rats was treated using isolated collagen extract for 15 days	Histochemical analysis revealed increasing of VEGF, TGF-β1 of the treated group	(Elbialy et al. 2020)
*Pinus brutia*	Bark	Proanthicin, catechin, epicatechin, and taxifolin	*In-vivo* model uses plant extract that is embedded in alginate gel dressing	The studied dressing showed a healing rate of 75.7% compared with control group with healing rate of 48.6%	(Karakaya et al. 2021)
*Pinus.pinaster*	Bark	Procyanidins, phenolic acids (ferulic and caffeic acids) and flavonoids (catechin, Taxifolin, quercetin and epicatechin)	Clinical trial was achieved with three groups of patients who suffered from venous ulcer and each group consists of six patients Group 1: received 50 mg of Pycnogenol ® (*P. pinaster*) orally three times daily Group 2: 50 mg of Pycnogenol ® (*P. pinaster*) orally three times daily + oral application of the drug on the ulcer Group 3: acted as control	Within 6 weeks, the oral + local treatment group showed faster healing, which was attributed to a significant increase in skin partial pressure of oxygen (PO2) and a decrease in skin partial pressure of carbondioxide (PCO2). Furthermore, there was a significant reduction in the ulcerated area	(Belcaro et al. 2005)
*Polygonum cuspidatum*	Aerial Part	Anthraquinone emodin, stilbene resveratrol, resveratrol glycoside, emodin methyl ether, and a few tannins and polysaccharides	*In-vivo* wound-healing model was used to assess the activity of extract and healing rates was reported at 3, 7, 14, and 21 days by immutohistochemical analysis	Treated group showed elevation of TGF-β1 that estimated by immutohistochemical analysis and increasing of fibroblasts accompanied with reduction of inflammatory cells	(Wu et al. 2012)
*Punica granatum*	Peel	Ellagitannins; punicalagin, ellagic acid, punicalin	*Punica granatum* peel gel was applied for 21 days on the wound that induced in diabetic rats	*P. granatum* had ability to regenerate collagen and enhance fibroblast infiltration that associated with elevation of TGF, VEGF and EGF	(Huan et al. 2013)
*Rheum officinale*	Root	Anthraquinone derivatives; emodin	Emodin Hydrogel 100, 200 and 400 μg/ml was evaluated against cutaneous wound of rats for 14 days	Emodin Hydrogel 400 μg/ml showed promising wound healing and tissue regeneration through TGF-β_1_ signaling pathway	(Tang et al. 2007)
*Sesamum indicum*	Seed oil	Sesamol	Sesamol was examined to know how it affects wound healing in albino rats, both in the normal and dexamethasone -delayed healing processes	These findings suggest that sesamol may be a useful medication for both normal and delayed wound healing	(Shenoy et al. 2011)
*Sambucus ebulus*	Leaf	Quercetin 3-O-glucoside	Circular excision and linear incision induced in rat and mice. Different plant fractions and isolated compound were formulated as ointments to heal different types of wounds	Methanolic fraction and isolated compound showed significant healing effect against different types of wounds	(Süntar et al. 2010)
*Sida rhombifolia Linn*	leaf	Alkaloids, saponin, tannin and sterols	Different concentration (25, 33, 50 and 80%) of plant extract ointment was used to heal Excision and incision wound in rats	The 50% ointment showed impressive fibrosis and collagenization	(Francis et al. 2018)
*Sphaeranthus indicus*	Aerial Part	Essential oil; Caryophyllene, flavonoids; Quercetin, Kaempferol, Sesquiterpenes; Sphaeranthine	Healing effect of plant cream extract was assessed on excised wounded model in Guinea pigs and compared with neomycin as control group	Plant cream enhanced the time of wound closure and suppressed the inflammatory markers	(Sadaf et al. 2006)
*Spirulina platensis*	Blue- green algae	Carbohydrates, protein, lipids and minerals	Algae were evaluated to treat wounds in rats for 21 days	Exerting antioxidant effects plays a great role in skin regeneration. Histological examination revealed that upregulating angiogenic bFGF and VEGF genes and occupying genes linked to scar formation, TGF-β and α-SMA	(Elbialy et al. 2021)
*Syzygium aromaticum*	Flower bud	Essential oil; eugenol, caryophyllene	*In-vivo* assay by applying nanoformulation of clove oil on wounded rats and compared with control group that treated with gentamycin	The formulation enhances the formation of leucine content, and the histological examination confirmed no inflammatory cell signals	(Alam et al. 2017)
*Thymus vulgaris*	Aerial part	Essential oil; thymol and carvacrol	Thyme oil with chitosan film dressing had assessed against different bacterial strains that affected wound	Significant Wound excision associated with antimicrobial activity *against Pseudomonas aeruginosa, Staphylococcus aureus, Escherichia coli, Klebsiella pneumoniae,*	(Altiok et al. 2010)
*Tridax procumbens*	Leaves	Flavonoids; (quercetin, kaempferol, and apigenin), Alkaloids; tridanine Triterpenoids; β-amyrin, Phenolic compounds; chlorogenic acid	*In-vitro* assay of Electrospun PCL Nanofibers of plant extract to analyze its microbial activity against bacterial infection that is associated with wounds	Nanofibers improve wound healing and repair surfaces contaminated by harmful germs	(Suryamathi et al. 2019)
*Vaccinium macrocarpon* (Cranberry)	Fruit	Anthocyanins	*In-vitro* models are based on using smart hydrogel film on wound and monitoring bacterial infection	Suppression of bacterial infection (Staphylococcus aureus, Pseudomonas aeruginosa)	(Zepon et al. 2019)
*Vernonia scorpioides*	Leaves	sesquiterpene lactones; scorpiolide, Scorpioidin	*In-vivo* assay using guinea pigs to estimate the plant extract hydrogel (200 mg with concentration 50%) for 30 days	The hydrogel enhanced the organization and regeneration of the new tissue but did not shorten the time of the closure	(Leite et al. 2002)
*Wedelia trilobata* (L.)	Leaves	Flavonoids, terpenoids, saponin and polyphenolics	*In-vitro* study to evaluate the effect of studied plants on L929 Fibroblasts cells to *Escherichia coli, Pseudomonas aeruginosa, Staphylococcus aureus and Staphylococcus epidermidis*	The plant provides promising wound-healing properties that associated with antibacterial effect and promoted L929 Fibroblasts cells	(Balekar, et al. 2012)
*Yucca aloifolia*	Seeds	Saturated Fatty acids; azelaic, stearic and palmetic Unsaturated fatty acids; linoleic and palmitoleic *γ*-sitosterol and phenolics	*In-vivo* study was designed to assess the effect of Yucca aloifolia seed gel and its nanoformulated gel	Significant suppress in biomarkers; TNF-α, TGF-β and VEGFA & with significar elevation in TNF-α and COX-2	(Bassam et al. 2024)
*Zataria multifora* Boiss	Aerial part	Essential oil; Thymol and Carvacrol	Nanogel of the plant oil was tried on wounded skin of rats for 21 days	Significant reduction of vascularity, inflammation, and edema with parallel increasing of fibrosis, re-epithelialization, and collagen deposition	(Osanloo et al. 2024)
*Zingiber officinale*	Rhizome	Oleoresin; gingerol, volatile oil and starch	Ginger assessed to treat incisional wound of rats	Ginger can improve the epithelialization of incisional wounds, decrease neutrophil counts, and raise fibroblast counts	(Rahayu et al. 2020)

In addition, the data from clinical trials demonstrating the efficacy of natural products for venous diseases were displayed in Table S1.

### Isolated natural compounds in venous disorders: anti-inflammatory effects, toxicity, and therapeutic potential

Diosmin and hesperidin are two citrus-derived flavonoids that have shown potent anti-inflammatory activities through the modulation of their inflammatory mediators (Harasstani et al. 2010). Diosmin has been reported to suppress NO, PGE2, and TNF-α production in lipopolysaccharide (LPS)-activated macrophages, indicating its anti-inflammatory activity (Harasstani et al. 2010; Abohashem et al. 2024). Comparisons with conventional anti-inflammatory drugs suggest that diosmin, particularly in association with other flavonoids, can possess comparable activity in inhibition of inflammatory processes. In addition, diosmin exhibits a good toxicity profile with low toxicity levels and protective effects against drug-induced organ injury, which is an attractive therapeutic implication (Harasstani et al. 2010; Wu et al. 2022).

Also, hesperidin has been shown to decrease NO, PGE2, TNF-α, and IL-6 production in inflammatory models (Choi et al. 2022). Although direct statistical comparison with traditional anti-inflammatory drugs is restricted, the anti-inflammatory potentiality of hesperetin, the aglycone form of hesperidin, is reported to be greater at low doses (Li et al. 2019b). In addition, hesperidin derivatives, such as hesperidin glucoside, maintain similar efficacy while offering improved solubility. The compound is typically safe with low level of acuteantub-chronic toxicity, but at high doses, slight physiological effects may occur. Given its anti-inflammatory effects and low cytotoxicity, hesperidin is a promising agent for therapeutic use (Li et al. 2019b).

Quercetin, a dietary flavonoid, is increasingly being studied for its possible therapeutic applications in the treatment of venous diseases (i.e., chronic venous insufficiency [CVI]. Its anti-inflammatory and antioxidant activities are at the core of its suggested role (Zhang et al. 2024).

In terms of inflammatory mediator production, quercetin has been shown to inhibit key pro-inflammatory cytokines such as TNF-α and IL-6. This inhibition is realized by blocking the JAK1/STAT3/HIF-1α signaling pathway, resulting in decreased inflammation and oxidative stress. These mechanisms are of relevance for venous pathologies in which chronic inflammation is a key factor in disease evolution (Miguel et al. 2025).

When compared to standard pharmacological treatments for CVI, such as diosmin and hesperidin, quercetin's efficacy in modulating inflammatory responses appears promising. However, direct statistical comparisons are limited. Pharmacological strategies for CVI under development have been tested with a view to clinical outcomes such as pain, oedema, and quality of life. Although quercetin's anti-inflammatory properties are well established, further systematic assessment is needed to confirm the comparative efficiency of quercetin compared to marketed venotonic agents (Huang et al. 2024).

Regarding toxicity, quercetin has been safely assumed with an advantageous profile. High doses in animal studies (up to 6 g/kg) did not result in organ damage or mortality. The subacute tests did not show any significant difference in histomorphometry, blood biochemistry, and blood counts. In human clinical studies, daily oral intake of 5000 mg of quercetin for 28 consecutive days in individuals with chronic hepatitis C did not lead to adverse effects.

Resveratrol, a polyphenolic compound present in grapes and red wine, has been studied for possible advantages in venous diseases on the basis of anti-inflammatory and antioxidant activities (Cottart et al. 2014). Evidence supports that resveratrol can also regulate inflammatory media such as the blocking of TNF-α and IL-6, that are central to the inflammatory process. Toxicity wise, resveratrol is [generally] well tolerated at low doses; however, at doses higher than 1 g/d, resveratrol can cause gastrointestinal problems, and paradoxically can cause hematological abnormalities on very rare occasions (Cottart et al. 2010). These results suggest that resveratrol could provide therapeutic potential for the treatment of venous conditions, but it is necessary to determine whether it is superior to conventional treatments (Meng et al. 2021).

Tranoiden saponins of *Centella asiatica*, Asiaticoside and Madecassoside, oleanane triterpenoid saponins, have been investigated on therapeutic effects of venous insufficiency (Tan et al. 2021). These compounds shows anti-inflammatory activity through inhibition of inflammatory mediators such as NO, TNF-α, and IL-6. Comparative analyses show that asiaticoside and madecassoide are able to reduce oxidative stress and inflammatory processes in different cell lines including macrophages and endothelial cells (Tan et al. 2021). In terms of toxicity, both compounds are regarded as safe but, some may have allergic reactions or intestinal upset. The effectiveness of their use in ameliorating signs and symptoms of venous insufficiency, including varicose veins has been validated by clinical practice, but direct statistical comparisons with conventional pharmacotherapy are sparse (Tan et al. 2021).

Ruscogenins, steroidal saponins isolated from *Ruscus aculeatus* (butcher's broom), have been used for the treatment of chronic venous insufficiency (Abascal and Yarnell 2002). It is thought that they have an anti-inflammatory effect by antagonizing elastase activity and by limiting vascular permeability resulting in reduction of edema and enhancement of venous tonicity (Bone 2003). While ruscogenins have been reported to be effective in alleviating symptoms such as leg swelling and discomfort, comprehensive data on their impact on specific inflammatory mediators and direct statistical comparisons with standard venotonic drugs are scarce (Lichota et al. 2019). Toxicity profiles indicate that ruscogenins are safe with rare reports of adverse events of clinical use (Ercan et al. 2020).

Ginkgolides and bilobalide, terpene lactones in *Ginkgo biloba* leaf extract, have been studied for their vascular effects (potentially with therapeutic use in venous system disorders) (Tian et al. 2017). These compounds show anti-inflammatory activity by blocking platelet-activating factor (PAF) and regulating cytokine production (Dziwenka and Coppock 2021). The clinical studies have found that ginkgolides can down regulate the expression of pro-inflammatory cytokines, which is related to enhancement of vasculoprotection (Silva and Martins 2022). Bilobalide also demonstrates neuroprotective and anti-inflammatory effects. Regarding toxicity *Ginkgo biloba* extracts are in general nontoxic if used properly but may interact with anticoagulant drugs and cause bleeding disorders (Liu et al. 2022).

### Commercially available preparations for venous diseases

Numerous products of biological origin have been developed in regions around the globe to help treat conditions relating to veins, like chronic venous insufficiency and varicose leg veins. Their healing properties and safety make these natural treatments stand out.

#### Miconized purified flavonoid fraction (MPFF)

MPFF is known under the trade mark Daflon. It is blended diosmin and hesperidin and derived from citrus fruits. It is meant for use in improvement of venous tone, inflammation and alleviation of symptoms of chronic venous disease (Awad et al. 2024; Lurie and Branisteanu 2023).

#### Horse chestnut seed extract

It is marketed as Venastaat or Aesculaforce. Horse chestnut seed extract contains aescin as its active ingredient. This extract is said to modify the tone of veins, decrease swelling of legs, and improve the venous inflow (Max and Pittler 2012).

#### Red vine leaf extract

Antistax is the commercial name under which it is sold. Red vine leaf extract is enriched with polyphenols such as quercetin and resveratrol. It is used to protects blood vessels, reduces edema, and enhances circulation (Stücker et al. 2019).

#### Gotu kola (Centella asiatica) extract

Sellers use the trademark Madecassol or Centellase. Gotu Kola extract is meant for healing wounds because it contains asiaticoside and madecassoside. It is meant also as an aid to microcirculation and to strengthen walls of veins (Park 2021).

#### Grape seed extract

Products containing Endotelon and Masquelier’s Original OPCs contain grape seed extract which carries oligomeric proanthocyanidin complexes. It is believed to have antioxidant capabilities and is also useful in improving venous tone along with alleviating the symptoms of venous insufficiency (Weseler and Bast 2017).

#### Ruscus aculeatus (Butcher’s broom) extract

Butcher’s broom extract is sold with the brand names Cyclo 3 Fort and Venoruton and contains ruscogenins. This herbal medicine is used for treating symptoms of chronic venous insufficiency like swelling and pain in the legs by improving venous tone as well as inflammation (Boyle et al. 2003).

### Ginkgo biloba extract

Tanakan and Tebonin are the marketed names for products containing Ginkgo Biloba extract, which consists of ginkgolides and bilobalide. Its vasodilator and antioxidant properties make it useful for blood flow and symptoms of various venous disorders (Kleijnen and Knipschild 1992).

#### Pycnogenol (Maritime pine bark extract)

The extract contains procyanidins and is sold under the name Pycnogenol. It strengthens the walls of capillaries, and helps to reduce swelling in the legs while improving circulation in people suffering from chronic venous insufficiency (Schoonees et al. 2012).

## Conclusions

CVDs are prevalent and progressive health issues, particularly affecting the aging population. Its incidence escalates with age, leading to a significant decline in quality of life due to symptoms associated with varicose veins and severe complications such as venous ulcers. Recent advances in the treatment of CVDs highlight the potential of natural products. Clinical studies have demonstrated that these products exert their effects through multiple mechanisms: they inhibit enzymes responsible for degrading the structural integrity of the venous wall, exhibit anti-inflammatory and antioxidant activities, possess vasoactive properties that enhance vascular tone, and have good ulcer healing potentials. Despite the therapeutic potential of these natural products, caution is warranted. The interaction between herbal extracts and conventional medications can lead to adverse effects, necessitating thorough investigation into their safety profiles. Furthermore, there is a pressing need for well-designed clinical trials that focus on specific formulations, dosages, and detailed compositions of these herbal remedies.

## Supplementary Information

Below is the link to the electronic supplementary material.

Supplementary file1 (DOCX 78 KB)

## Data Availability

All data are available within the manuscript.

## Abbreviations

AI: Artificial intelligence
BMI: Body mass index
BRBNS: Bleb Nevus Syndrome
CEAP: Clinical, etiological, anatomical, and pathophysiological
CVD: Cardiovascular disease
CVI: Chronic venous insufficiency
CVMs: Congenital venous malformations
DVT: Deep venous thrombosis
EDS: Ehlers-Danlos syndrome
EVLA: Endovenous laser ablation
KTS: Klippel-Trenaunay syndrome
LDS: Lymphedema distichiasis syndrome
MPFF: Micronized purified flavonoid fraction
OPCs: Oligomeric proanthocyanidins
PE: Pulmonary thromboembolism
PTA: Percutaneous transluminal angioplasty
PTS: Post-thrombotic syndrome
PWS: Parkes Weber syndrome
RFA: Radiofrequency ablation
SVT: Superficial venous thrombosis
VV: Varicose veins
