# Supplementary material for: Therapeutic applications of natural products in the management of venous diseases: a comprehensive review
Source: Inflammopharmacology. 2025 Mar 12;33(4):1673–712. doi: 10.1007/s10787-025-01688-z (PMC11992006; doi:10.1007/s10787-025-01688-z)
Supplement: Supplementary file 1 — Supplementary file1 (DOCX 78 KB) [file 10787_2025_1688_MOESM1_ESM.docx]

**Table S1.** Clinical trials data demonstrating the efficacy of natural products in venous disorders.

| No. | Plant name | Type of extract / compound | Part used | Family | Methods | Action | Treatment | Methods data | Side effects | Ref. |
| --- | --- | --- | --- | --- | --- | --- | --- | --- | --- | --- |
| 1 | *Ginkgo biloba* | Ethanol | Leaves | Ginkgoaceae | In vitro and in vivo models | Improves circulation, decreases platelet aggregation, and blood clotting. | Blood Clots, DVT, Thrombophlebitis | Ginkgo biloba extract enhances the effectiveness of cilostazol. It has been proposed that GB inhibits platelet aggregation by increasing cAMP, inhibiting the formation of thromboxane A2, and activating MMP9.  Specifically, the combination of cilostazol (25 mg/kg) and GB (20 mg/kg) increased the survival rate more than double the dose of each drug alone. | Bleeding | (Ryu et al., 2009; Li et al., 2019) |
| 2 | *Aesculus hippocastanum* | Water | Seeds | Sapindaceae | Clinical Trials | Reduces swelling, strengthens veins and improves venous tone in CVI. | Chronic Venous Insufficiency | In 240 individuals with a diagnosis of CVI, leg compression stockings were compared to an aesculus and placebo. 300 mg of an aesculus extract that was equivalent to 50 mg of aescin. Aesculus extract was administered twice a day.  The average amount of leg edema in CVI patients was calculated to be 220 milliliters.  Nine subjects received a placebo after 12 weeks of treatment, whereas 10 participants received 300 mg of an aesculus extract twice daily. The findings demonstrated that, in comparison to a placebo, the aesculus extract prevented a swelling differential of about 60 ml in every patient. | Allergic dermatitis, headaches, nausea, and gastrointestinal distress | (Zampieron, 2017; Bencsik et al., 2024) |
| 3 | *Curcuma longa* | Ethanol | Rhizomes | Zingiberaceae | In vitro | Curcumin has anti-coagulant activity. | Blood Clots, DVT | 100 μl of an ethanolic extract of Curcuma caesia rhizomes and streptokinase was administered to the positive control tube, while distilled water was introduced to the negative control alpine tube. while distilled water. Which was used as a negative control, exhibited very little clot lysis (2.96±0.28%), the extract showed 49.18±3.41% clot lysis, and the addition of 100 μl streptokinase had 71.54±3.26% clot lysis. | - | (Fathima et al., 2015; McEwen, 2015) |
| 4 | *Centella Asiatica* | Water | Leaves | Apiaceae | Clinical Trials, Lab Studies | Improves blood vessel integrity and relieves symptoms of varicose veins. | Chronic Venous Insufficiency | At a double-blind, placebo-controlled research study, 87 patients with chronic venous hypertension microangiopathy received oral standardized C. asiatica product at two dosages (30 mg and 60 mg), which improved microcirculatory parameters in a dose-dependent way as compared to placebo. demonstrated that C. asiatica can lower arterial blood pressure in hypertensive individuals in addition to venous insufficiency. When 4 g of gotu kola tea was administered three times a day, this was noted. | Hypersensitivity reactions and headache | (Udombhornprabha, 2018; Bencsik et al., 2024) |
| 5 | *Capsicum frutescens* | Aqueous | Fruits | Solanaceae | In vitro | Increases blood flow, dilates vascular structures, and may even prevent coagulation. | DVT, Varicose Veins | The clot lysis activity of the Capsicum frutescens extract was 40.65%. The clots exhibited 78.23% clot lysis when 100 µl of streptokinase, a standard thrombolytic medication, was added as a positive control. However, clots treated with 100 µl of sterile distilled water (negative control) only displayed a very small amount of clot lysis activity (3.52%). | - | (Arifuzzaman et al., 2011; Rhone et al., 2018) |
| 6 | *Taraxacum officinale* | Water | Root | Asteraceae | In vitro | Acts as a diuretic and improves circulation, thus relieving the symptoms of CVI. | DVT | Platelet aggregation was decreased by about 40–50% at the maximum dose (1 mg/mL). This implies that Taraxacum might have a slight anticoagulant effect, which might potentially influence the production of clots in vascular disorders such as DVT. | - | (López-Pérez et al., 2022) |
| 7 | *Trifolium pratense* | Methanol | Flower heads | Fabaceae | Clinical Trials, Lab Studies | Promotes circulation and tones up blood vessels; good for varicose veins and venous insufficiency. | Blood Circulation, Varicose Veins | The anti-adhesive actions of Trifolium phenolics were assessed in comparison to the control (untreated) platelets, which adhesion was assumed to be 100%, while the different concentrations of T. pratense extracts: 1, 5, and 50 (µg/ml) resulted in 90.72±3.05, 84.40±3.43, and 81.21±4.48, respectively. | - | (Kolodziejczyk-Czepas et al., 2016; Mokhtari et al., 2020) |
| 8 | *Acorus calamus* | Ethanol | Rhizomes | Acoraceae | In vitro | Improves circulation and may reduce the risk of clotting. | Venous Circulation | To a microcentrifuge tube containing a pre- weighed clot, 100 μl of ethanolic extract of P. nigrum and A. calamus at varying concentrations (200 mg, 400 mg, and 800 mg) was added individually. At 200 mg, 400 mg, and 800 mg of A. calamus, 100 μl of streptokinase (76.11) and 100 μl of distilled water (7.69) were applied separately as positive and negative controls, respectively, for the percentage of clot lysis (53.96, 51.66, and 48.79). | - | (Emon et al., 2020) |
| 9 | *Pinus pinaster* | Supercritical CO2 | Bark | Pinaceae | Clinical Trials, Animal Studies | Pycnogenol increases circulation, defends veins, and minimizes swelling. | Blood Clots, DVT | The increased platelet reactivity and aggregation observed 2 h after smoking were inhibited by a single dose of either 500 mg of acetyl salicylic acid or 100 mg of Pycnogenol. This inhibitory effect on platelet reactivity followed a clear dose–response relationship, producing a complete absence of platelet aggregation at a dose of 200 mg of Pycnogenol, the effect persisting for 6 days. The effects of Pycnogenol on platelet aggregation were also confirmed after repeated oral administration. Seven heavy smokers were given Pycnogenol at a daily dose of 200 mg for 8 weeks. The platelet aggregation index was significantly reduced at the end of the trial. | - | (Gulati, 2014) |
| 10 | *Mangifera indica* | Ethanol | Leaves | Anacardiaceae | Animal Studies | Blood flow maintained; prevents clotting through the strengthening of vein walls. | Blood Circulation, Antioxidant Support | Aqueous-methanolic leaf extract of M. indica at 20%, 10%, and 5% dilutions results in 65.81 + 1.37, 41.52 + 1.42, and 18.62 + 1.31, when compared to the standard drug, streptokinase 78.71 + 1.48. | - | (Ain, 2022a, b) |
| 11 | *Vitis vinifera* | Ethanol | Seeds | Vitaceae | Clinical Trials, Lab Studies | Grape seed extract has antioxidants and may prevent clot formation. | Blood Clots, Thrombophlebitis | According to a randomized, double-blind, crossover study, postmenopausal women's blood pressure and vascular endothelial function improved after taking 300 mg of grape seed extract supplements for four weeks. | Gastrointestinal disorders, headache, and hypersensitivity reactions | (Bencsik et al., 2024) |
| 12 | *Ruscus aculeatus* | Methanol | Root | Ruscaceae | Clinical Trials | Reduces symptoms of chronic venous insufficiency, including swelling and leg heaviness. | Blood Circulation, CVI | The findings of 20 randomized, double-blind, placebo-controlled investigations; 5 randomized studies versus a positive control, and 6 single-arm studies including 10,246 patients were compiled in a meta-analysis. In comparison to a placebo, an oral combination medication (150 mg of R. aculeatus root extract per capsule, 150 mg of hesperidin methyl chalcone, and 100 mg of ascorbic acid) used to treat chronic vein disease may lessen the intensity of discomfort, cramping, heaviness, and paranesthesia. Additionally, venous capacity was significantly decreased in comparison to a placebo. | It is not advised to use during pregnancy or lactation. | (Bihari et al., 2022; Bencsik et al., 2024) |
| 13 | *Trema orientalis* | Different extracts | Leaves | Cannabaceae | In vitro | Blood flow maintained, prevents clotting | Blood clots | After 90 minutes of incubation at 37°C and the addition of 100 μL SK (positive control) to the clots, 80.77 ± 1.12% clot lysis was observed. Clots treated with sterile distilled water (negative control) only showed 5.69±3.09% clot lysis, which is extremely low. The positive and negative control groups' mean clot lysis percentage differences were highly significant (P values <0.001 and 0.05, respectively). Additionally, T. orientalis chloroform fractions (46.44%) demonstrated considerable (P values < 0.001) clot lysis. However, T. orientalis hydro-methanol fractions have considerable clot lysis activity (45.78%), while T. orientalis ethyl acetate fractions have significant clot lysis activity (43.29%) with P values <0.001. | - | (Emran et al., 2015) |
| 14 | *Urena sinuata* | Different extracts | Leaves | Malvaceae | In vitro | Blood flow maintained, prevents clotting | Blood clots | After 90 minutes of incubation at 37°C and the addition of 100 μL SK (positive control) to the clots, 80.77 ± 1.12% clot lysis was observed. Clots treated with sterile distilled water (negative control) only showed 5.69±3.09% clot lysis, which is extremely low. The positive and negative control groups' mean clot lysis percentage differences were highly significant (P values <0.001 and 0.05, respectively). Additionally, U. sinuata chloroform fractions (47.89%) demonstrated considerable (P values < 0.001) clot lysis. while U. sinuata ethyl acetate fractions have significant clot lysis activity (38.29%) with P values < 0.05. | - | (Emran et al., 2015) |
| 15 | *Cinnamomum verum* | Ethanol | Bark | Lauraceae | In vitro | Improves circulation and blood flow and reduces blood viscosity. | Venous Health, Blood Circulation | It has long been known that cinnamonaldehyde inhibits blood platelet aggregation in vitro. proved that this substance prevented platelet aggregation brought on by thrombin and collagen in vitro and, for the first time, revealed its inhibitory effects on platelet aggregation in vivo. Additionally, these authors noted that giving this chemical to animals stops platelet-related thrombosis. Another cinnamon component, eugenol, inhibits thromboxane A2 to decrease platelet aggregation. | - | (Raja et al., 2020; Das et al., 2022) |
| 16 | *Crocus sativus* | Ethanol | Stigma | Iridaceae | In vitro | Saffron has anti-coagulant properties, which reduce blood clotting. | DVT, Blood Clots | These investigations demonstrated Foxo3a's contradictory involvement in thrombosis. Here, we found that patients with LEDVT had higher levels of Foxo3a. Furthermore, PIM1 positively regulated Foxo3a, which it targeted. The effects of PIM1 knockdown were reversed by overexpressing Foxo3a, indicating that the PIM1/Foxo3a axis was the underlying mechanism of crocin-regulated angiogenesis and apoptosis as well as Foxo3a's thrombosis-promoting actions. | - | (Chen et al., 2023) |
| 17 | *Peucedanum japonicum* | coumarin derivative, 3′,4′-Diisovalerylkhellactone diester (PJ-1) | - | Umbelliferae | In vivo | Enhances blood flow and exhibits slight anticoagulant properties, thereby supporting overall cardiovascular well-being. | Blood Clots, DVT | 3′,4′-Diisovalerylkhellactone diester (PJ-1), a coumarin derivative that was isolated from medicinal P. japonicum, has been demonstrated to prevent ATP release and rabbit platelet aggregation brought on by PAF (IC50=56.3 μM) or collagen (IC50=89.4 μM). Furthermore, PJ-1 decreased the production of TXB2 brought on by collagen. PJ-1 also prevented the phosphoinositide degradation and intracellular Ca2+ rise brought on by PAF. Additionally, with an IC50 value of 3.9 μM, PJ-1 prevented PAF from binding to cleaned platelets. | - | (El Haouari and Rosado, 2016) |
| 18 | *Withania somnifera* | Ethanol | Root | Solanaceae | Clinical Studies | Enhances blood flow and exhibits slight anticoagulant properties, thereby supporting overall cardiovascular well-being. | DVT, Blood Clots | WFA significantly prolonged a PTT and PT at doses of 1 μM and beyond, while having lesser anticoagulant properties than heparin or warfarin. | - | (Ku and Bae, 2014; Basudkar et al., 2024) |
| 19 | *Pistacia chinensis* | Methanol | - | Anacardiaceae | In vitro | Blood flow maintained, prevents clotting | Blood Clots, Thrombophlebitis | ADP-induced platelet aggregation was reduced by P. chinensis methanolic extract (PCME) at concentrations ranging from 2.5 to 20 μg/mL. Additionally, PCME increased cAMP synthesis in resting platelets and decreased [Ca2+]i, ATP, and TXA2 creation in ADP-activated platelets. Additionally, PCME decreased Akt, JNK, and ERK phosphorylations and prevented fibrinogen from binding to αIIbβ3. Because of its putative antiplatelet properties, P. chinensis may be used for cardiovascular disease prevention and/or treatment. | - | (El Haouari and Rosado, 2016) |
| 20 | *Allium sativum* | Ethanol | Bulb | Amaryllidaceae | Clinical Trials, Lab Studies | Garlic improves circulation and has anti-clotting activity. | Blood Clots, Varicose Veins | Randomized clinical trial Evaluation of platelet aggregation and bleeding time after three weeks of 600, 1200, and 2400 mg garlic pill administration in healthy males, in comparison to 75 mg Plavix. Platelet aggregation induced by ADP and AA agonists decreased by garlic (1200 or 2400 mg). The bleeding time increased in those who received a 2400 mg garlic pill. | Bleeding | (Alaraky, 2018) |
| 21 | *Boswellia serrata* | Chloroform | Resin | Burseraceae | Clinical Studies | Anti-inflammatory and blood circulation-enhancing effects. | Blood Clots, Thrombophlebitis | Heparin, the positive drug control in this investigation, prevented ADP-induced platelet aggregation. In a concentration-dependent manner, BSAE and BSWE significantly reduced platelet aggregation. High antiplatelet aggregatory activity was demonstrated by both extract types, BSAE and BSWE (3 mg dwt/mL), with a p value <0.001. The extracts' activity was similar to that of heparin. Thus, our findings showed that BS gum resin has an antiplatelet aggregatory action. | - | (Kokkiripati et al., 2011; Valente et al., 2024) |
| 22 | *Ocimum basilicum* | Aqueous | Leaves and stalks | Lamiaceae | In vivo | Reduction in thrombus weight | DVT | Six groups of 60 Wistar rats were randomly assigned: three OBL groups at varying concentrations (15, 75, and 375 mg/Kg), aspirin, and control. For fifteen days in a row, the animals received oral treatment with the corresponding medicines.  Rats given an aqueous extract of OBL orally for two weeks saw a dose-dependent reduction in thrombus weight in the in vivo arterio-venous shunt thrombosis paradigm. | - | (Tohti et al., 2006) |
| 23 | *Cinnamomum cassia* | Ethanol | Bark | Lauraceae | Clinical Studies | It improves circulation and possesses some anticoagulant properties. | DVT, Blood Clots | It has long been known that cinnamon aldehyde inhibits blood platelet aggregation in vitro. proved that this substance prevented platelet aggregation brought on by thrombin and collagen in vitro and, for the first time, revealed its inhibitory effects on platelet aggregation in vivo. Additionally, these authors noted that giving this chemical to animals stops platelet-related thrombosis. Another cinnamon component, eugenol, inhibits thromboxane A2 to decrease platelet aggregation. | - | (Chase et al., 2022; Das et al., 2022) |
| 24 | *Piper nigrum* | Ethanol | Fruit | Piperaceae | Clinical Studies | Improves circulation and may reduce the risk of clotting. | Venous Circulation | To a micro centrifuge tube containing a pre weighed clot, 100 μl of ethanolic extract of P. nigrum and A. calamus at varying concentrations (200 mg, 400 mg, and 800 mg) was added individually. To determine the percentage of clot lysis at 200 mg, 400 mg, and 800 mg of P. nigrum, 100 μl of Streptokinase (76.11) and 100 μl of distilled water (7.69) were applied separately as positive and negative controls, respectively. | - | (Emon et al., 2020; Jain et al., 2023) |
| 25 | *Crataegus orientalis* | Ethanol | Leaves | Rosaceae | In vivo | Improves circulation and may reduce the risk of clotting. | Venous Circulation, Blood Clots | The results of the current investigation showed that COE greatly reduced the in vivo tail thrombosis caused by carrageenan in mice, and that the amount of COE injected boosted the inhibitory activity. When compared to control groups throughout the 24-72-hour period when findings were gathered, 100 mg/kg COE and 10 IU heparin did not significantly alter outcomes. Both 200 and 300 mg/kg of COE demonstrated a significant antithrombotic effect at the 24-hour mark (pb0.05 and pb0.01, respectively). | - | (Arslan et al., 2011) |
| 26 | *Zingiber officinale* | Ethanol | Rhizomes | Zingiberaceae | Clinical Trials, Lab Studies | Has good properties in improving circulation, ant-inflammatory, and blood-thinning. | Blood Circulation, Venous Health | In order to ascertain the impact of ginger extract on platelet function, the current study examined how it affected bleeding time. The difference in bleeding time between the groups under investigation was statistically significant (p=0.03).  The aspirin (positive control) had a mean of 141.4, but the high dose group (HDG) had a mean of 139.2. There were notable differences between these two groups and the others.  The bleeding time rose in a dose-dependent way with the greatest (130.6) and lowest (127.2) readings for MD and 5% DMSO, respectively, even though there was no statistically significant difference between the low dose (LD), medium dose (MD), and 5% DMSO. | - | (McEwen, 2015; Shadrack et al., 2019) |
| 27 | *Salvia miltiorrhiza* | Methanol | Root | Lamiaceae | Clinical Studies | Known to enhance circulatory dynamics and relieve venous edema and pain. | CVI, Varicose Veins | Effects of a week of Salvia miltiorrhiza intravenous infusion. (A) The Salvia group's prothrombin time was slower than that of the control group (*P<0.05). (B-D) The other coagulation function assessments, such as activated partial prothrombin, fibrinogen, and thrombin timings, did not show any statistically significant changes (P>0.05). | - | (Cao et al., 2015) |
| 28 | *Euphorbia neriifolia* | Ethanol | Whole plant | Euphorbiaceae | In vivo | Known for its anti-inflammatory and mild anticoagulant properties, which help in blood flow and prevent clots. | Blood Circulation, DVT | After receiving an intravenous injection of carrageenan, swelling and redness were noticed two to three hours later. After six hours, the tail turned auburn, suggesting that thrombosis had developed there. The average length of the infarcted area in the rats' tails was 10.1 ± 0.6 cm in rats treated with 200 mg/kg extract, 9.2 ± 0.3 cm in rats treated with 400 mg/kg extract, and 6.5 ± 0.7 cm in rats treated with heparin. The group treated with saline experienced an infarction of approximately 11.1 ± 0.3 cm. The control group did not experience any infarction. | - | (Hasan et al., 2014) |
| 29 | *Sesamum indicum L* | Isopropanol | Seeds | Pedaliaceae | In vitro | Fibrinolytic activity | Blood clots | Broke down the fibrin clot to remove blood fibrinogen. | - | (Liu and Chiang, 2008) |
| 30 | *Carica papaya* | Ethanol | Leaves | Caricaceae | Clinical Studies | Improves circulation and reduces swelling associated with venous disease. | Blood Circulation, CVI | In Sprague-Dawley rats, papain inhibits thrombosis caused by κ-carrageenan.  Papain and streptokinase had inhibitory effects on the formation of tail thrombus at 48 hours. The data are presented as follows: The control group's thrombus length was 11.6±0.4 cm, the SK (2000) U length was 8 cm, and the various papain dosages were 2.5, 5, and 10 (U/kg) and 8.5, 6, and 2.5, respectively. | - | (Koehler et al., 2022; Yang et al., 2023) |
| 31 | *Citrus sinensis* | Ethanol | Peel, Fruit | Rutaceae | Animal Studies | Orange peel, which is high in flavonoids and antioxidants, enhances blood flow and decreases venous swelling. | Blood Circulation, Varicose Veins | Rabbits were administered C. sinensis juice at three different dosages: 2 ml/kg, 5 ml/kg, and 8 ml/kg. All three doses resulted in a considerable rise in the erythrocyte count, while the doses of 5 and 8 milliliters per kilogram were found to considerably boost hemoglobin. Prothrombin time was significantly prolonged at 5 ml/kg and 8 ml/kg, but thrombin time and activated partial thromboplastin time were significantly prolonged at all doses, comparable to warfarin. Protein C levels increased significantly at 5 and 8 milliliters per kilogram, while the thrombin antithrombin complex increased significantly at 8 milliliters per kilogram. Only epinephrine significantly reduced the percentage of platelets that aggregated at 2 ml/kg, but at 5 ml/kg, platelet aggregation was inhibited. | - | (Mallick and Khan, 2014; Leite et al., 2022) |
| 32 | *Carthamus tinctorius* | Ethanol | Flowers | Asteraceae | Clinical Studies | Reduces inflammation and supports proper blood circulation; used commonly for venous insufficiency. | Venous Health, Circulation | The rats were split up into six groups: reference group (aspirin 5 mg/kg), normal group, control group, and groups that got 20, 40, and 80 mg/kg of CTL, respectively. For 14 days, each group received therapy orally once a day. All dosages of CTL extracts showed substantial and dose-dependent differences from the control group. enhanced inhibition rate, decreased thrombus weight, and extended thrombosis occlusion time (p < 0.01). | - | (Wu et al., 2014; Ding et al., 2015) |
| 33 | *Hypericum perforatum* | Ethanol | Flowers | Hypericaceae | Clinical Trials, Animal Studies | Known for its anti-inflammatory and mild anticoagulant properties, which help in blood flow and prevent clots. | Venous Circulation, Blood Clots | We discovered that HP exhibits antiplatelet properties in vitro, and that the inhibitory effect is closely correlated with the quantities employed. Following HP treatment, there was a significant decrease in intracellular Ca++ mobilization responses and activated GP IIb IIIa (fibrinogen receptors) expression, leading to an inhibition of over 50%. | - | (Scholz et al., 2021; Monteiro et al., 2022) |
| 34 | *Morus alba* | Methanol | Leaves | Moraceae | In vivo | Antiplatelet activity | Venous Circulation, Blood Clots | ADP was inhibited by collagen-induced platelet aggregation, thrombin-induced platelet aggregation did not limit TXA2 production and ADP, serotonin release was inhibited, and fibrinogen binding was inhibited by Ca2+mobilization. | - | (Kim et al., 2014) |
| 35 | *Nigella sativa* | Aqueous extract | Seeds | Ranunculaceae | In vitro | Reduce swelling and promote circulation in conditions such as DVT and varicose veins with thrombolytic activity | Blood Circulation, DVT | The clot lysis activity of the Nigella sativa extract was 36.93%. The clots exhibited 78.23% clot lysis when 100 µl of Streptokinase, a well-known thrombolytic medication, was added as a positive control. However, clots treated with 100 µl of sterile distilled water (negative control) only displayed a very small amount of clot lysis activity (3.52%). | - | (Arifuzzaman et al., 2011) |
| 36 | *Gloriosa superb* | - | Leaves | Lilaceae | In vivo | decreases the fibrin clot formation | Blood clots | With an IC50 value of 2.97 mg/ml, the leaf extracts demonstrated anticoagulant qualities by preventing thrombin-induced coagulation. | - | (Kee et al., 2008) |
| 37 | *Porana volubilis* | - | Leaves and flowers | Convolvulaceae | In vitro | anticoagulant activity | Blood clots | Heparin cofactor II, not antithrombin, mediates the improvement of thrombin inhibition, which in turn mediates its anticoagulant action. Heparin cofactor II mediates the augmentation of thrombin inhibition, which in turn mediates anticoagulant action. | - | (Yoon et al., 2002) |
| 38 | *Plumbago zeylanica* | Ethanol | Root, Leaf | Plumbaginaceae | Animal Studies | Historically used to improve blood circulation, it has mild anticoagulant properties. | DVT, Blood Circulation | The results of the trial indicate that MEPZ at three test concentrations (100, 200, and 300 mg/kg) had an in vivo thrombolytic effect at 24 and 48 hours (p<0.001). At 24 and 48 hours, the normal group's average tail thrombosis lengths were 12.40±1.36 and 12.36±1.12, respectively. Following treatment with 100, 200, and 300 mg/kg of MEPZ, the length of the tail thrombosis decreased to 9.64±1.45, 8.63±1.61, and 7.96±1.67 at 24 hours and 9.36± 1.97, 7.69±0.39, and 7.03±1.07 at 48 hours, respectively. | - | (Guguloth et al., 2022; Guguloth et al., 2023) |
| 39 | *Mentha spicata* | Mthanol and chloroform | Leaves | Lamiaceae | In vitro | Known for its property to cause vasodilation that increases blood flow and reduces swelling in the lower limbs. | Varicose Veins, Circulation | Effects of medications on the breakdown of clots made from healthy people's blood. The highest clot lysis (75. ± 3.04%) was seen in the SK-treated clot. When M. spicata was exposed to methanol, its clot lysis activity was 30.89%, and when it was exposed to chloroform, it was 29.77%. The clot lysis rate in water (as a negative control) was 3.29%. | - | (Shahik et al., 2014) |
| 40 | *Mentha arvensis* | Mhanol and chloroform | Leaves | Lamiaceae | In vitro | Known for its property to cause vasodilation that increases blood flow and reduces swelling in the lower limbs. | Blood Clots, DVT | Effects of medications on the breakdown of clots made from healthy people's blood. The highest clot lysis (75. ± 3.04%) was seen in the SK-treated clot. When M. arvensis was exposed to methanol, its clot lysis activity was 32.56%, and when it was exposed to chloroform, it was 31.87%. The clot lysis rate in water (as a negative control) was 3.29%. | - | (Shahik et al., 2014) |
| 41 | *Mentha viridis* | Mthanol and chloroform | Leaves | Lamiaceae | In vitro | Known for its property to cause vasodilation that increases blood flow and reduces swelling in the lower limbs. | Blood Circulation, DVT | Effects of medications on the breakdown of clots made from healthy people's blood. The highest clot lysis (75. ± 3.04%) was seen in the SK-treated clot. When M. viridis was exposed to methanol, its clot lysis activity was 30.29%, and when it was exposed to chloroform, it was 29.77%. The clot lysis rate in water (as a negative control) was 3.29%. | - | (Shahik et al., 2014) |
| 42 | *Rheum palmatum* | Ethanol | Root | Polygonaceae | Clinical Studies | Reduces inflammation, improves healthy blood circulation, and may be beneficial in preventing the formation of venous thrombus. | Venous Health  , Circulation | Chrysophanol-8-O-glucoside (CP8-O-GLC) was discovered to have the most potent inhibitory impact on platelet aggregation caused by thrombin and collagen.  By preserving the levels of NO and ET-1, which expand and constrict blood vessels, rhubarb may have the effect of increasing blood circulation and alleviating blood stasis. | - | (Yang et al., 2017; Zhou and Lei, 2023) |
| 43 | *Glycyrrhiza glabra* | Ethanol | Root | Fabaceae | In vivo | Licorice has an anti-inflammatory action that reduces swelling and enhances venous circulation. | DVT, Thrombophlebitis | The mean thrombus weight was 9.1F2.0 mg (n=8), and 100% of thrombus occurrence was observed in a control group that received tissue thromboplastin dosages of 3 mg/kg. With an ED50 of 75 mg/kg, GL administered intravenously resulted in a progressive reduction in thrombus weight, illustrates the time-dependent pattern of GL's antithrombotic effect in the stasis model. following 5 minutes of drug delivery, GL doses of 180 mg/kg provided 93% inhibition; however, 60 minutes following drug administration, the same GL dose only demonstrated 35% inhibition when the thrombogenic stimulus was delivered. | hypokalemia, water and salt retention, and hypertension | (Mendes-Silva et al., 2003; Shi et al., 2020) |
| 44 | *Cichorium intybus* | Ethanol | Root | Asteraceae | In vitro | Promotes blood flow, reduces swelling, and is good for the treatment of varicose veins. | Varicose Veins, Blood Circulation | In vitro, caffeine prevents platelet aggregation. Additionally, we have corroborated this finding:  In vitro collagen-induced platelet aggregation was reduced by 50% by 1.2 mM caffeic acid (data not shown). Following consumption of cory coffee, the 10 µM ADP-induced platelet aggregation increased at 2 hours and on day 8 as well. There was no discernible difference in the platelet aggregation caused by 5 µM ADP. At two hours, collagen-induced platelet aggregation was lower than baseline, whereas adrenaline-induced aggregation was higher. | - | (Khosropanah et al., 2023) |
| 45 | *Cucurbita pepo* | Ethanol | Seed, Fruit | Cucurbitaceae | In vivo | Rich in antioxidants, pumpkin improves circulation and may help reduce venous inflammation. | Blood Circulation, DVT | The effect of Cucurbita pepo seed extract on PT (shortened PT and increased PC following similar with the control) suggests that there is a link between PT and PC, in contrast to heparin-induced rats that demonstrated a considerable extension of PT and a significant reduction of PC. | Bleeding | (Harenberg, 2008; Agbai and Nwanegwo, 2013) |
| 46 | *Aloe vera* | Water | Leaf Gel | Asphodelaceae | Clinical Studies | Aloe vera is used for its anti-inflammatory properties, which reduce swelling in the legs and improve circulation. | Blood Circulation, Varicose Veins | The mean platelet inhibition with AVL was roughly 71.54%, and the mean platelet aggregation was 25.01%. The mean platelet aggregation and inhibition with AVH were 16.29% and 81.5%, respectively compared to aspirin mean platelet inhibition 95.39%.   Aloe vera was therefore shown to reduce platelet aggregation in a dose-dependent manner. In comparison to celecoxib's mean platelet inhibition of -1.87%, aspirin, AVH, and AVL all statistically significantly decreased platelet aggregation%. | Bleeding | (Udombhornprabha, 2018; Mushtaq et al., 2020) |
| 47 | *Rosmarinus officinalis* | Ethanol | Leaves, Oil | Lamiaceae | Clinical Trials | Rosemary "Improves blood circulation and can lower clotting," thanks to its mild anti-coagulant properties. | Blood Clots, DVT | Applying rosemary ointment topically promotes local circulation, generates heat, and reduces inflammation and pain. In clinical research including 46 patients in intensive care units, the anti-inflammatory properties of a topical ointment made from rosemary were shown in the case of phlebitis brought on by antibiotic medication. Eucerin® (8.25 g) and rosemary essential oil (4.2 g) make up the rosemary ointment (30 g). Both preclinical research and traditional use point to the successful topical treatment of R. officinalis for mild circulatory problems, despite the lack of strong clinical proof. | It is not advised to use during pregnancy or lactation. | (Bencsik et al., 2024) |
| 48 | *Brassica oleracea* | Aqueous extract | Whole plant | Brassicaceae | In vitro | Supports venous health by promoting greater blood flow and reducing inflammation in the vascular system. with thrombolytic activity | Venous Health, DVT | The clot lysis activity of the Brassica oleracea extract was 57.03%. The clots exhibited 78.23% clot lysis when 100 µl of Streptokinase, a well-known thrombolytic medication, was added as a positive control. However, clots treated with 100 µl of sterile distilled water (negative control) only displayed a very small amount of clot lysis activity (3.52%). | - | (Arifuzzaman et al., 2011) |
| 49 | *Fucus vesiculosus* | Ethanol | Seaweed | Fucaceae | In vivo | May help improve blood circulation and support venous health due to its iodine and antioxidant properties. | Blood Circulation, Varicose Veins | The concentration necessary to double the total occlusion time was determined to be the antithrombotic action (effective dosage, 50% [ED50]). Heparin's and fucoidan's ED50 values were roughly 1.24 and 0.54 mg/kg body weight, respectively. Consequently, in vivo, fucoidan demonstrated an antithrombotic activity that was almost 2.3 times stronger than heparin. | - | (Kwak et al., 2010; Chandika et al., 2022) |
| 50 | *Butea monosperma* | Ethanol | Bark, Leaves | Fabaceae | In vitro | To increase the circulation of blood, minimize edema, and thrombi formation. | Blood Clots, DVT | When tested for thrombolytic activity against a positive control SK, the CME and extractives of B. monosperma leaves demonstrated 65.15 ± 0.16% clot lysis. However, the negative control, sterile distilled water, showed a very small percentage of clot lysis (8.20 ± 0.16%). PSF (27.36 ± 0.23%), AQFS (21.64 ± 0.25%), CTSF (20.74 ± 0.43%), CSF (19.90 ± 0.43%), and CME (12.88 ± 0.33%) were the fractions that showed the highest percentages of clot lysis. Considering >20% moderate, it may be inferred from the data that the leaf extract of B. monosperma showed moderate thrombolytic activity. | - | (Jarald et al., 2009; Uddin et al., 2017) |

**References**

Agbai, E., and C. Nwanegwo. 2013. EFFECT OF Cucurbita Pepo ON PROTHROMBIN TIME AND PLATELET COUNT IN HEPARIN-INDUCED THROMBOCYTOPENIC ALBINO WISTAR RATS. 5: 24 – 31.

Ain, Q. U. 2022a. Anticoagulant and thrombolytic activities of leaf extract of mangifera indica in smokers. Tobacco Regulatory Science (TRS): 1189-1201.

Ain, Q. U. 2022b. Anticoagulant and Thrombolytic Activities of Leaves Extract of Mangiferara Indica. Tobacco Regulatory Science (TRS): 250-258.

Alaraky, N. E. E. 2018. Effect of Allium Sativum (Garlic) Intake on Prothrombin Time and International Normalize Ratio, Omkalthoum Osman Hamad.

Arifuzzaman, M. et al. 2011. Evaluation of thrombolytic properties of Nigella sativa, Capsicum frutescens and Brassica oleracea. International Journal of Research in Pharmaceutical Sciences 2: 483-487.

Arslan, R., Z. Bor, N. Bektas, A. H. Meriçli, and Y. Ozturk. 2011. Antithrombotic effects of ethanol extract of Crataegus orientalis in the carrageenan-induced mice tail thrombosis model. Thrombosis Research 127: 210-213.

Basudkar, V. et al. 2024. Emerging Vistas for the Nutraceutical Withania somnifera in Inflammaging. Pharmaceuticals 17: 597.

Bencsik, T. et al. 2024. Herbal drugs in chronic venous disease treatment: An update. Fitoterapia: 106256.

Bihari, I., J.-J. Guex, A. Jawien, and G. Szolnoky. 2022. Clinical Perspectives and Management of Edema in Chronic Venous Disease—What about Ruscus? Medicines 9: 41.

Cao, H. et al. 2015. Salvia miltiorrhiza prevents deep vein thrombosis via antioxidative effects in endothelial cells. Molecular medicine reports 11: 3593-3600.

Chandika, P. et al. 2022. Marine biological macromolecules and chemically modified macromolecules; potential anticoagulants. Marine Drugs 20: 654.

Chase, C., A. Doyle, S. St John, T. Laurent, and S. Griffith. 2022. Post-operative haemorrhage secondary to cinnamon use. A case report. International Journal of Surgery Case Reports 95: 107179.

Chen, J., Y. Yang, S. Wang, and K. Zhang. 2023. Crocin improves lower extremity deep venous thrombosis by regulating the PIM1/FOXO3a axis. Cellular and Molecular Biology 69: 183-188.

Das, G. et al. 2022. Cardiovascular protective effect of cinnamon and its major bioactive constituents: An update. Journal of Functional Foods 97: 105045.

Ding, T.-b. et al. 2015. Ethanol extract of Carthamus tinctoriusL. shows anti-thrombosis activity in rats. African Journal of Traditional, Complementary and Alternative Medicines 12: 120-124.

El Haouari, M., and J. A. Rosado. 2016. Medicinal plants with antiplatelet activity. Phytotherapy Research 30: 1059-1071.

Emon, N. U. et al. 2020. Anxiolytic and thrombolytic investigation of methanol extract of Piper nigrum L. fruits and Sesamum indicum L. seeds. J. Adv. Biotechnol. Exp. Ther 3: 158-164.

Emran, T. B. et al. 2015. Effects of organic extracts and their different fractions of five Bangladeshi plants on in vitro thrombolysis. BMC complementary and alternative medicine 15: 1-8.

Fathima, S. N., S. V. Ahmad, and B. R. Kumar. 2015. Evaluation of in vitro thrombolytic activity of ethanolic extract of Curcuma caesia rhizomes. International Journal of Pharma Research & Review 4: 50-54.

Guguloth, S. K., N. Malothu, N. M. Ganta, K. Ramakrishna, and C. Guntupalli. 2023. Antiplatelet and antithrombotic properties of methanolic leaf extract of plumbago zeylanica L.: GC-MS and HR-LCMS metabolite profiling. South African Journal of Botany 159: 627-634.

Guguloth, S. K., N. Malothu, D. Prasanth, and A. R. Areti. 2022. Evaluation of the Thrombolytic and Antioxidant Activity of Leaf Extracts of Plumbago zeylanica L. INDIAN JOURNAL OF PHARMACEUTICAL EDUCATION AND RESEARCH 56: 1181-1189.

Gulati, O. P. 2014. Pycnogenol® in chronic venous insufficiency and related venous disorders. Phytotherapy research 28: 348-362.

Harenberg, J. 2008. Drugs affecting blood coagulation, fibrinolysis, and hemostasis Side Effects of Drugs Annual No. 30. p 399-422. Elsevier.

Hasan, M., A. Ganeshpurkar, D. Bansal, and N. Dubey. 2014. Protective effect of Euphorbia neriifolia extract on experimentally induced thrombosis in murine model. Nigerian Journal of Experimental and Clinical Biosciences 2: 86-89.

Jain, V., B. Kunwar, and S. Verma. 2023. A Review on Thrombolysis Enhancing Indian Edible Plants. Biomedical and Pharmacology Journal 16: 1283-1302.

Jarald, E., N. Narendra, M. Manish, J. Anurekha, and E. Sheeja. 2009. Determination of rutin content and antioxidant activity of extracts of Butea monosperma flowers extracted using various extraction methods. Phcog J 1: 126-129.

Kee, N. L. A., N. Mnonopi, H. Davids, R. J. Naudé, and C. L. Frost. 2008. Antithrombotic/anticoagulant and anticancer activities of selected medicinal plants from South Africa. African Journal of Biotechnology 7.

Khosropanah, A. et al. 2023. Effects of chicory and fumitory on hot flashes among breast cancer survivors: a randomized, double-blind placebo-controlled trial. Journal of Integrative and Complementary Medicine 29: 31-41.

Kim, D.-S. et al. 2014. Antiplatelet activity of Morus alba leaves extract, mediated via inhibiting granule secretion and blocking the phosphorylation of extracellular‐signal‐regulated kinase and akt. Evidence‐Based Complementary and Alternative Medicine 2014: 639548.

Koehler, A. et al. 2022. A case study using papaya leaf extract to reverse chemotherapy-induced thrombocytopenia in a GBM patient. Integrative Cancer Therapies 21: 15347354211068417.

Kokkiripati, P. K. et al. 2011. Gum resin of Boswellia serrata inhibited human monocytic (THP-1) cell activation and platelet aggregation. Journal of ethnopharmacology 137: 893-901.

Kolodziejczyk-Czepas, J. et al. 2016. The anti-adhesive and anti-aggregatory effects of phenolics from Trifolium species in vitro. Molecular and cellular biochemistry 412: 155-164.

Ku, S.-K., and J.-S. Bae. 2014. Antiplatelet, anticoagulant, and profibrinolytic activities of withaferin A. Vascular pharmacology 60: 120-126.

Kwak, K.-W. et al. 2010. Biological effects of fucoidan isolated from Fucus vesiculosus on thrombosis and vascular cells. The Korean journal of hematology 45: 51.

Leite, P. M. et al. 2022. In vitro anticoagulant activity of selected medicinal plants: Potential interactions with warfarin and development of new anticoagulants. Journal of Basic and Clinical Physiology and Pharmacology 33: 499-510.

Li, J., Q. Liang, and G. Sun. 2019. Interaction between traditional Chinese medicine and anticoagulant/antiplatelet drugs. Current Drug Metabolism 20: 701-713.

Liu, B.-L., and P.-S. Chiang. 2008. Production of hydrolysate with antioxidative activity and functional properties by enzymatic hydrolysis of defatted sesame (Sesamum indicum L.). International Journal of Applied Science and Engineering 6: 73-83.

López-Pérez, A. et al. 2022. Laelia furfuracea Lindl.: an Endemic Mexican Orchid with Anticoagulant Activity. Journal of the Mexican Chemical Society 66: 1-16.

Mallick, N., and R. A. Khan. 2014. Effect of Citrus sinensis (Sweet Orange) on coagulation. South Asian J. Exp. Biol 4: 54-60.

McEwen, B. J. 2015. The influence of herbal medicine on platelet function and coagulation: a narrative review. In: Seminars in Thrombosis and Hemostasis. p 300-314.

Mendes-Silva, W. et al. 2003. Antithrombotic effect of Glycyrrhizin, a plant-derived thrombin inhibitor. Thrombosis research 112: 93-98.

Mokhtari, M. et al. 2020. The efficacy of topical red clover oil on knee osteoarthritis: A pilot prospective randomized triple‐blind placebo‐controlled clinical trial. Phytotherapy Research 34: 1687-1695.

Monteiro, M.-d.-C., A. C. Dias, D. Costa, A. Almeida-Dias, and M. B. Criado. 2022. Hypericum perforatum and its potential antiplatelet effect. In: Healthcare. p 1774.

Mushtaq, S. et al. 2020. Comparison of effect of aloe Vera gel with aspirin and celecoxib on platelet aggregation. The Professional Medical Journal 27: 973-978.

Raja, A., B. Varalakshmi, and K. Santhi. 2020. Antibacterial, Antioxidant and Anticoagulant Efficacy of C. verum Mediated Silver Nanoparticles.

Rhone, P. et al. 2018. Comprehensive analysis of haemostatic profile depending on clinicopathological determinants in breast cancer patients. Bioscience reports 38: BSR20171657.

Ryu, K. H. et al. 2009. Ginkgo biloba extract enhances antiplatelet and antithrombotic effects of cilostazol without prolongation of bleeding time. Thrombosis Research 124: 328-334.

Scholz, I. et al. 2021. Effects of Hypericum perforatum (St John's wort) on the pharmacokinetics and pharmacodynamics of rivaroxaban in humans. British journal of clinical pharmacology 87: 1466-1474.

Shadrack, K., A. Faraj, M. K. Alex, and N. Kenneth. 2019. Anti-thrombotic effect of Zingiber officinale (ginger) in Sprague Dawley rats. Int J Res Med Sci 7: 3239.

Shahik, S. M. et al. 2014. In vitro thrombolytic and cytotoxic evaluation of Mentha arvensis L., Mentha spicata L. and Mentha viridis L. Journal of Pharmaceutical and Biological Sciences 9: 97-102.

Shi, C.-C. et al. 2020. Inhibition of human thrombin by the constituents of licorice: Inhibition kinetics and mechanistic insights through in vitro and in silico studies. RSC advances 10: 3626-3635.

Tohti, I. et al. 2006. Aqueous extracts of Ocimum basilicum L.(sweet basil) decrease platelet aggregation induced by ADP and thrombin in vitro and rats arterio–venous shunt thrombosis in vivo. Thrombosis research 118: 733-739.

Uddin, J., A. J. Bishwash, and Z. K. Labu. 2017. Preventive activities against thrombosis and inflammation of Butea monosperma (Lam.) leaves methanolic extract in vitro model. Am. J. Res. Med. Sci 2: 58-65.

Udombhornprabha, A. 2018. The efficacy and safety of herbal medicine combination for management of leg symptoms due to chronic venous diseasethai patients: a double blind ed.

Valente, I. V. B. et al. 2024. The anti-proliferative effects of a frankincense extract in a window of opportunity phase ia clinical trial for patients with breast cancer. Breast Cancer Research and Treatment 204: 521-530.

Wu, S.-h. et al. 2014. Anti-thrombotic effect of Carthamus tinctorius Linn extracts in rats. Tropical Journal of Pharmaceutical Research 13: 1637-1642.

Yang, H. R. et al. 2023. Unveiling the potent fibrino (geno) lytic, anticoagulant, and antithrombotic effects of papain, a cysteine protease from carica papaya latex using κ-carrageenan rat tail thrombosis model. International Journal of Molecular Sciences 24: 16770.

Yang, T.-z. et al. 2017. The use of Rheum Palmatum L. in the treatment of acute respiratory distress syndrome: a meta-analysis of randomized, controlled trials. African Journal of Traditional, Complementary and Alternative medicines 14: 334-347.

Yoon, S.-J. et al. 2002. The medicinal plant Porana volubilis contains polysaccharides with anticoagulant activity mediated by heparin cofactor II. Thrombosis research 106: 51-58.

Zampieron, E. 2017. Horse chestnut (Aesculus hippocastanum) for venous insufficiency. Int J Complement Alt Med 5: 00153.

Zhou, X., and G. Lei. 2023. Research Progress on Modern Pharmacological Action of Rhubarb. MEDS Chinese Medicine 5: 138-146.
